# Sound Absorption Modeling in Porous Materials: A Critical Review of Empirical, Equivalent-Fluid, Poroelastic, Resonant, and Numerical Methods

**DOI:** 10.3390/ma19153207

**Published:** 2026-07-27

**Authors:** Marek Nikodym, Martin Vasina

**Affiliations:** 1 Faculty of Electrical Engineering and Computer Science, VŠB-Technical University of Ostrava, 17. listopadu 2172/15, 708 00 Ostrava-Poruba, Czech Republic; 2 Faculty of Mechanical Engineering, VŠB-Technical University of Ostrava, 17. listopadu 2172/15, 708 00 Ostrava-Poruba, Czech Republic

**Keywords:** porous materials, sound absorption, acoustic modeling, equivalent-fluid models, empirical models, poroelastic models, Johnson–Champoux–Allard model, Biot theory

## Abstract

This paper provides a comprehensive overview of the main empirical, equivalent-fluid, poroelastic, resonant, and numerical models used to describe sound absorption in porous materials. Each model is described in detail with regard to its theoretical basis, governing equations, and key physical parameters. Special attention is devoted to the assumptions underlying each model, such as whether the frame is rigid or flexible, the applicable frequency range, and the types of porous media they most accurately represent. The advantages and limitations of the different approaches are critically assessed in terms of prediction accuracy, computational complexity, physical interpretability, and experimental requirements. In addition, this paper summarizes and critically discusses published model–experiment comparisons for representative porous and resonant acoustic materials. These comparisons highlight the strengths and weaknesses of different modeling strategies in various acoustic applications and provide guidance for selecting the most suitable model according to the material properties and target frequency range. The review shows that equivalent-fluid models generally provide the best compromise between prediction accuracy and computational efficiency for rigid-frame porous materials, whereas Biot-type poroelastic models are more suitable when frame motion cannot be neglected.

## 1. Introduction

Noise is considered one of the most widespread environmental stressors. Environmental noise is the second largest contributor to disability adjusted life years lost in Europe, after air pollution [1]. It reduces well-being and quality of life, disrupts interpersonal communication and mental concentration, and induces emotional responses corresponding to noise pollution [2]. Excessive noise can lead not only to hearing loss but also to cognitive impairment, sleep disturbances, gastrointestinal disorders, endocrine dysfunction, psychophysiological effects, and cardiovascular disease [3]. Therefore, it is essential to reduce noise pollution through appropriate control measures. In general, noise control strategies are categorized into active and passive methods [4]. The aim of active noise control (ANC) technology is to attenuate undesired acoustic noise by generating an anti-noise signal of equal amplitude and opposite phase, thereby achieving destructive interference between the two signals [5]. In contrast, passive noise control (PNC) systems utilize sound-absorbing materials that reduce noise by dissipating acoustic energy and converting it into heat [6].

In terms of sound absorption mechanisms, acoustic absorbers can be classified into two main categories: porous absorbers, which attenuate sound through viscous friction and thermal losses within their pore structures, and resonant absorbers, which utilize acoustic resonance to dissipate sound energy [7]. Porous absorbers are further subdivided according to their microstructure into fibrous, foam (cellular), and granular materials [8]. The ability of open-porous material structures to dampen sound is closely related to their airflow resistance [9]. In general, increasing airflow resistance enhances sound absorption across the entire frequency range, but only up to an optimal value. If the material becomes too acoustically resistant (i.e., if the airflow resistance is excessively high), its sound absorption decreases due to the limited penetration of acoustic waves into the porous structure. Resonant absorbers [7,10] include Helmholtz resonators, flexible plates or membranes, and micro-perforated panels, among others. Each of these sound-absorbing materials exhibits optimal performance in different frequency ranges. Conventional porous absorbers (e.g., mineral wool and polymer foams) are particularly effective at absorbing high-frequency sound. However, their absorption efficiency decreases significantly in the mid- and low-frequency ranges. In contrast, resonant absorbers can effectively absorb low-frequency sound, although usually only in a narrow band around their resonance frequency [11]. This complementarity means that no single material alone can provide broadband sound absorption, as the limiting factor is the characteristic wavelength of sound. To achieve effective sound absorption at a given frequency, the thickness of the absorber typically needs to be approximately one quarter of the wavelength (*λ*/4) [12]. However, at low frequencies where wavelengths are long, such thicknesses become both dimensionally impractical and economically unfeasible. As a result, resonant elements or combinations of different materials [13] are often employed in low-frequency sound absorption applications.

In addition to conventional natural fibers and agricultural by-products, recent research has focused on new sustainable sound-absorbing materials. One of the emerging areas of research involves hydroponically grown, bio-based porous composites, in which plant root systems form a natural binding network that stabilizes the porous substrate after drying. Iannace et al. [14] demonstrated that such materials, prepared from wheat grown on various renewable substrates, can achieve normal-incidence sound absorption coefficients approaching unity in selected mid-frequency bands. These materials combine promising acoustic performance with the use of renewable or recycled resources.

For the reasons mentioned above, many researchers are investigating multilayer and hybrid systems that combine porous and resonant mechanisms to increase absorption efficiency over a wider frequency range. For example, layered sandwich structures incorporating different porous materials, gradient porosity, or combinations of porous media and cavity resonators can achieve higher absorption coefficients, particularly in the mid- and low-frequency bands, than a single homogeneous layer. Zhang et al. [15] reported in their review of fibrous structures that combining multiple absorption mechanisms, namely porous damping, material damping and resonance, enhances absorption at low frequencies while maintaining effective absorption at high frequencies. Similarly, SheikhMozafari et al. [16] demonstrated that combining a conventional fibrous absorber with a micro-perforated panel can extend the absorption bandwidth toward higher frequencies. However, a non-optimal design may, conversely, reduce sound absorption in certain frequency ranges, highlighting the importance of careful parameter selection and layer configuration.

Acoustic metamaterials represent a distinct class of sound absorbers. These are artificially engineered structures that enable the manipulation of sound and elastic waves beyond the capabilities of conventional materials. They are composed of periodic or quasi-periodic subwavelength units that generate local resonances, resulting in extraordinary properties such as negative effective density, negative bulk modulus, or anisotropic sound propagation [17,18,19]. These unique properties allow acoustic metamaterials to effectively absorb, control, or focus sound. For example, additively manufactured (3D-printed) metamaterial absorbers with carefully arranged perforations, slits, or resonant cavities can exhibit multiple absorption peaks, covering a broad frequency range [20,21]. Acoustic metamaterials thus open the way to the design of thin, highly effective absorbers tailored to a specific frequency range. Recent research has increasingly focused on hybrid acoustic metamaterials that combine porous media with locally resonant structures, micro-perforated panels, or labyrinth cavities [22,23]. Such hybrid structures significantly improve low-frequency sound absorption while maintaining compact dimensions, thereby overcoming one of the main limitations of conventional porous absorbers. Recent advances in additive manufacturing have enabled these complex geometries to be fabricated with high precision, making hybrid metamaterials increasingly attractive for practical engineering applications [22,24].

In recent years, the development of mathematical simulations has become an important trend in various areas of research, including the analysis of frequency dependencies of the normal-incidence sound absorption coefficient. These simulations provide valuable tools for predicting and interpreting experimental results.

The aim of this work is to critically review and compare the main empirical, equivalent-fluid, poroelastic, resonant, and numerical models used to describe sound absorption in porous materials. The study focuses on identifying the key parameters and assumptions that influence the accuracy and applicability of individual models under different acoustic conditions. Another objective is to assess the ability of selected models to reproduce experimental results through numerical simulations and published validation studies. Particular emphasis is placed on the practical interpretation of model assumptions, the comparison of prediction capabilities across different classes of porous materials, and the identification of conditions under which increased model complexity leads to improved predictive performance. The findings provide a structured basis for selecting appropriate modeling approaches according to material characteristics, target frequency range, and the required balance between accuracy, computational cost, and experimental effort.

## 2. Theoretical Background: Definition of the Sound Absorption Coefficient and Its Measurement Methods

### 2.1. Sound Absorption Coefficient

The ability of a material to absorb sound is expressed by the sound absorption coefficient *α*, which is defined by the equation [25]:

(1) $$\alpha =\frac{P_{a}}{P_{i}}=\frac{P_{i}-P_{r}}{P_{i}}=\frac{P_{d}+P_{t}}{P_{i}}$$

where *P_a_* is the absorbed acoustic power, *P_i_* is the incident acoustic power, *P_r_* is the reflected acoustic power, *P_d_* is the dissipated acoustic power inside the tested sample, and *P_t_* is the transmitted acoustic power.

### 2.2. Measurement Methods for the Sound Absorption Coefficient

In general, there are three basic methods for measuring the sound absorption coefficient:

- Acoustics—Measurement of sound absorption in a reverberation room according to ISO 354 [26].
- Acoustics—Determination of sound absorption coefficient and impedance in impedance tubes. Part 1: Method using standing wave ratio according to ISO 10534-1 [27].
- Acoustics—Determination of sound absorption coefficient and impedance in impedance tubes. Part 2: Transfer function method according to ISO 10534-2 [28].

#### 2.2.1. Measurement of Sound Absorption in a Reverberation Room

The reverberant sound absorption coefficient is defined as the ratio:

(2) $$\alpha =\frac{A_{T}}{S}$$

where *A_T_* is the equivalent sound absorption area of the tested sample, and *S* is the area of the tested sample. The equivalent sound absorption area of the tested sample is expressed by the equation:

(3) $$A_{T}=A_{2}-A_{1}=55.3V\left(\frac{1}{c_{2}T_{2}}-\frac{1}{c_{1}T_{1}}\right)-4V\left(m_{2}-m_{1}\right)$$

where *A*_1_ and *A*_2_ are the sound absorption areas in the empty room and in the room with the tested sample, *V* is the volume of the reverberation room, *c*_1_ and *c*_2_ are the propagation speeds of sound in air in the room without and with the tested sample, *T*_1_ and *T*_2_ are the reverberation times of the empty reverberation room and after the test specimen has been installed, and *m*_1_ and *m*_2_ are the power attenuation coefficient of the climatic conditions in the reverberation room without and with the tested sample determined according to ISO 9613–1 [29]. The measurement of the sound absorption coefficient in a reverberation room is performed on samples with an area greater than 10 m^2^ and utilizes a diffuse sound field that simulates actual acoustic conditions in rooms. However, this procedure is time-consuming and costly because it requires a special reverberation room with a minimum volume of 150 m^3^ [26].

#### 2.2.2. Measurement of Sound Absorption Coefficient in Impedance Tubes Using Standing Wave Ratio

In this method, a plane acoustic wave is generated inside a rigid cylindrical impedance tube and propagates toward a sample placed at one end. Part of the wave is absorbed by the material, while the remaining part is reflected, creating a standing wave due to interference between the incident and reflected waves. The maximum and minimum acoustic pressures (i.e., *p_max_* and *p_min_*) in the standing wave are measured along the axis of the tube using a movable microphone [27]. The absolute value of the reflection coefficient *r* is then calculated using the following formula:

(4) $$\left|r\right|=\frac{\left|p_{max}\right|/\left|p_{min}\right|-1}{\left|p_{max}\right|/\left|p_{min}\right|+1}$$

Finally, the normal-incidence sound absorption coefficient *α* is determined as:

(5) $$\alpha =1-{\left|r\right|}^{2}$$

If the acoustic pressures are measured on a logarithmic scale (in decibels) and the sound pressure level difference between the pressure maximum and minimum is **Δ***L*, then the sound absorption coefficient can alternatively be expressed as:

(6) $$\alpha =\frac{4\cdot 10^{∆L/20}}{{\left(10^{∆L/20}+1\right)}^{2}}$$

The standing wave ratio method (SWR) is simple, cost-effective, and suitable for laboratory conditions. It allows testing of small material samples and does not require complex instrumentation. However, it is applicable only to plane (normally incident) sound waves and therefore does not reflect real acoustic conditions in rooms. Moreover, it is limited by its frequency range, and manual measurement of pressure maxima and minima is prone to errors.

#### 2.2.3. Measurement of Sound Absorption Coefficient in Impedance Tubes Using Transfer Function Method

The transfer function method [28] is similar to the SWR method in many respects. It utilizes normal sound incidence on the sample, as well as a comparable impedance tube configuration and specimen preparation procedure. However, instead of a single movable microphone, it utilizes two fixed microphones, which enable the determination of the complex acoustic transfer function between the two measurement positions.

Based on this method, the normal-incidence sound absorption coefficient is calculated from the reflection factor according to Equation (5), where the normal-incidence reflection factor *r* is determined from the transfer function method as:

(7) $$r=\frac{H_{12}-H_{I}}{H_{R}-H_{12}}\cdot e^{2k_{c}x_{1}j}$$

where *H*_12_ is the complex acoustic transfer function, *H_I_* is the transfer function for the incident wave, *H_R_* is the transfer function for the reflection wave, *k_c_* is the complex wavenumber, and *x*_1_ is the distance between the sample surface and the farther microphone M_1_. The transfer functions are expressed as follows:

(8) $$H_{12}=\frac{p_{2}}{p_{1}}=\frac{e^{k_{c}x_{2}j}+r\cdot e^{{-k}_{c}x_{2}j}}{e^{k_{c}x_{1}j}+r\cdot e^{{-k}_{c}x_{1}j}}$$

(9) $$H_{I}=e^{{-k}_{c}\left(x_{1}-x_{2}\right)j}$$

(10) $$H_{R}=e^{k_{c}\left(x_{1}-x_{2}\right)j}$$

where *p*_1_ and *p*_2_ are the complex sound pressures at the two microphone positions, and *x*_2_ is the distance between the sample surface and the nearer microphone M_2_.

The normal-incidence reflection factor *r* can also be expressed by the equation [30,31]:

(11) $$r=\frac{Z_{s}-Z_{0}}{Z_{s}+Z_{0}}=\frac{Z_{s}-\rho_{0}c_{0}}{Z_{s}+\rho_{0}c_{0}}$$

where *Z_s_* is the surface acoustic impedance, *Z*_0_ is the characteristic impedance of air, *ρ*_0_ is the air density, and *c*_0_ is the sound speed in the air. The surface acoustic impedance is given by the equation:

(12) $$Z_{s}=-jZ_{c}cot(k_{c}t)$$

where *Z_c_* is the characteristic impedance, and *t* is the material thickness. The characteristic impedance and the complex wavenumber are determined via the frequency-dependent effective density (*ρ*(*ω*)) and bulk modulus (*K*(*ω*)) that account for viscous and thermal diffusion in a pore space [26]:

(13) $$Z_{c}=\sqrt{\rho \left(\omega \right)K\left(\omega \right)}$$

(14) $$k_{c}=\omega \cdot \sqrt{\frac{\rho \left(\omega \right)}{K\left(\omega \right)}}$$

where *ω* is the angular frequency (*ω* = 2*π·f*), and *f* is the frequency.

Overall, the transfer function method has largely superseded the SWR method in modern impedance tube measurements because it is faster, suitable for automated digital measurements, and provides direct complex transfer function data. The method is widely applied in research on the acoustic properties of materials, in the verification of numerical models, and in quality control in industrial applications.

It should be noted that the absorption coefficients obtained from impedance tubes and reverberation rooms correspond to different acoustic conditions. Impedance tube measurements provide normal-incidence absorption coefficients, whereas reverberation room measurements provide diffuse-field absorption coefficients. Therefore, comparisons between measured values should always consider the applied measurement method.

## 3. Mathematical Models for Simulation of Sound Absorption Behavior

This chapter presents an overview of the main mathematical models used to simulate the frequency dependence of the sound absorption coefficient. It describes their basic assumptions, input data requirements, advantages, limitations, and typical applications, with particular emphasis on the factors affecting prediction accuracy and practical applicability. Most analytical models provide a frequency-dependent description of the absorber’s acoustic behavior, from which the reflection coefficient and sound absorption coefficient are calculated using the impedance formulation presented in Section 2.2.3.

Based on their physical basis, the approaches discussed in this chapter are classified into empirical models, semi-empirical and equivalent-fluid models, fully physical poroelastic models, resonant analytical models, and numerical or hybrid approaches. Empirical formulations are based primarily on experimentally derived relationships and provide effective solutions when only limited information about the material is available. Equivalent-fluid approaches account for viscous and thermal phenomena at the pore level through effective material parameters, while poroelastic formulations additionally consider the mechanical response of the solid frame. Resonant and numerical approaches are considered for structures whose acoustic behavior cannot be adequately described by conventional porous-layer formulations. In addition to constitutive descriptions of porous and resonant absorbers, computational frameworks and inverse identification methods used for numerical implementation, parameter estimation, and model optimization are also briefly described.

### 3.1. Empirical Models

Empirical models express the characteristic impedance and complex wavenumber directly as fitted functions of frequency and airflow resistivity. Unlike physically based approaches, they do not explicitly describe the viscous and thermal boundary layer effects within a porous medium. Instead, the influence of material morphology is incorporated into experimentally derived coefficients obtained from impedance tube measurements. Their main advantage is simplicity, as they typically require only one or a few material parameters, which allows for their direct use in impedance tube analysis, transfer matrix calculations, and preliminary absorber design. Their main limitation is restricted transferability, as the fitted coefficients apply primarily to classes of materials and morphological conditions similar to those used during calibration.

Once the characteristic impedance and complex wavenumber are obtained from the empirical formulations, they are introduced into the impedance formulation to determine the surface impedance, reflection factor, and subsequently the sound absorption coefficient. Therefore, empirical models differ mainly in the way the frequency-dependent acoustic properties of the absorber are estimated from measurable material parameters.

#### 3.1.1. General Delany–Bazley-Type Empirical Form

Most empirical models can be expressed using a generalized power-law formulation based on the Delany–Bazley approach [31]. In this formulation, the characteristic impedance and complex wavenumber are written as:

(15) $$Z_{c}=\rho_{0}c_{0}[1+C_{1}X^{C_{2}}-jC_{3}X^{C_{4}}]$$

(16) $$k_{c}=\frac{\omega }{c_{0}}[1+C_{5}X^{C_{6}}-jC_{7}X^{C_{8}}]$$

where *X* is the dimensionless frequency-resistivity parameter defined as:

(17) $$X=\frac{\rho_{0}f}{\sigma }$$

where *σ* is the airflow resistivity. The coefficients *C*_1_–*C*_8_ define the individual empirical formulations and are applied according to the sign convention adopted in Equations (15) and (16). Their validity ranges and corresponding coefficient values are summarized in Table 1.

This generalized formulation provides a compact representation of several empirical approaches developed for porous sound absorbers. While most models follow the power-law form given by Equations (15) and (16), some formulations use alternative mathematical expressions. For comparison, Table 1 also includes the Komatsu model. However, its coefficients are source-specific coefficients for the logarithmic expressions given by Equations (18) and (19), not generic power-law coefficients; therefore, they must not be substituted directly into Equations (15) and (16).

#### 3.1.2. Delany–Bazley Model

The Delany–Bazley model is a classical single-parameter empirical model for highly porous fibrous sound absorbers. It was derived from impedance tube measurements of glass-fiber and mineral-wool materials with porosities approaching unity. Within the notation used in this article, the model is obtained by inserting the Delany–Bazley coefficients from Table 1 into Equations (15) and (16). The model is particularly useful for rapid preliminary calculations when airflow resistivity is the only reliably known non-acoustical parameter [31,34].

The main advantages of the Delany–Bazley model are its minimal parameter requirements, low computational cost, and straightforward coupling with rigid backing conditions, air gaps, and multilayer transfer matrix formulations. Its limitations include a restricted calibration range, reduced accuracy at low frequencies, and the fact that it does not explicitly take into account porosity, tortuosity, characteristic lengths, pore size distribution, or elastic-frame effects. Therefore, it is most suitable for mineral wool, glass wool, and other highly porous fibrous materials, while its application to granular media, closed-cell foams, architected lattices, resonant absorbers, or limp-frame materials requires additional validation.

#### 3.1.3. Mechel Model

Mechel’s model was introduced to extend the applicability of Delany–Bazley-type formulations to lower values of *X* and to address the unphysical negative real part of the surface impedance that can occur at low frequencies [35]. For the ranges (1/60 < *X* < 1) and (*X* ≤ 1/60), the model retains the general form of Equations (15) and (16), but the low-frequency coefficients must be copied with the same sign convention as the source formulation; the corrected coefficients are listed in Table 1. This model is advantageous in cases where a Delany–Bazley-type model is required, but the frequency range extends toward lower frequencies or to coarser fibrous structures. However, it remains an empirical approach that should be used only within its range of validity and in accordance with the sign convention adopted in the source equations.

#### 3.1.4. Miki Model

The Miki model retains the algebraic structure of the Delany–Bazley formulation but modifies the coefficients to ensure that the impedance satisfies the positive-real condition and that the propagation constant exhibits more physically consistent behavior. The corresponding coefficients are listed in Table 1 and are substituted into Equations (15) and (16). This correction is important in multilayer transfer matrix calculations, where non-physical impedance predictions, such as negative resistance terms, can lead to unrealistic absorption peaks or numerical instability [36].

This model is widely used for fibrous materials, natural fibers, and highly porous lightweight absorbers when detailed microstructural data are not available. Previous research has shown that Miki-type predictions can provide reasonable accuracy for a wide range of fibrous materials. However, the model remains a single-parameter empirical formulation and cannot distinguish between materials with the same airflow resistivity but different pore size distributions, tortuosity, or frame stiffness [31].

#### 3.1.5. Garai–Pompoli Model

The Garai–Pompoli model is a specialized empirical formulation developed for polyester fiber blankets. It retains the same general formulation as Equations (15) and (16), but uses coefficients optimized for polyester blankets with fiber diameters of approximately 18–48 μm. The model was calibrated by minimizing the deviation between measured and calculated sound absorption coefficients and therefore illustrates the advantage of material-specific empirical calibration [37].

Its main advantage is potentially improved accuracy for polyester nonwovens compared with generic coefficient sets. Its main limitation is correspondingly limited transferability, requiring independent validation when applied to wool, cellulose, coconut fiber, glass wool, or foam-based materials.

#### 3.1.6. Komatsu Model

Komatsu’s model extends the Delany–Bazley and Miki model families by introducing a logarithmic dependence on the frequency-to-resistivity ratio. It therefore does not follow the power-law form used in Equations (15) and (16). Within the notation used in this article, Komatsu’s logarithmic expressions are written with the source convention using the common logarithm log_10_ as follows:

(18) $$Z_{c}=\rho_{0}c_{0}[1+C_{1}{\left(2-\log_{10}\frac{f}{\sigma }\right)}^{C_{2}}-j\;C_{3}{\left(2-\log_{10}\frac{f}{\sigma }\right)}^{C_{4}}]$$

(19) $$k_{c}=\frac{\omega }{c_{0}}[1+C_{5}{\left(2-\log_{10}\frac{f}{\sigma }\right)}^{C_{6}}-j\;C_{7}{\left(2-\log_{10}\frac{f}{\sigma }\right)}^{C_{8}}]$$

This model can improve prediction accuracy for denser fibrous materials and for low-density materials that fall outside the optimal range of the original Delany–Bazley model. Its disadvantages include the empirical nature of the logarithmic correction, sensitivity to the implementation conventions for the common logarithm log_10_ and the *f*/*σ* ratio, and reduced robustness when applied to multilayer or highly heterogeneous systems unless validated against experimental data [38].

#### 3.1.7. Voronina Model

For fibrous porous media, the Voronina model introduces a structural parameter defined as a function of porosity and fiber diameter, thereby relating the acoustic response to this morphology-sensitive descriptor rather than relying solely on airflow resistivity [31,39,40]. The model is expressed by the following equations:

(20) $$Z_{c}=\rho_{0}c_{0}[1+Q-jQ]$$

(21) $$k_{c}=\frac{\omega }{c_{0}}[\frac{Q\left(2+Q\right)}{1+Q}+j\left(1+Q\right)]$$

(22) $$Q=\frac{\left(1-\phi \right)\left(1+q_{0}\right)}{\phi \cdot d}\sqrt{\frac{4\eta }{\pi f\rho_{0}}}$$

(23) $$q_{0}=\frac{1}{1+2\cdot 10^{4}\cdot {\left(1-\phi \right)}^{2}}\;$$

where *Q* is the structural characteristic, *ϕ* is the porosity, *d* is the fiber diameter, *η* is the air dynamic viscosity coefficient, and *q*_0_ is the parameter related to the effect of fiber motion on acoustical behavior.

After performing several modifications, Voronina presented an improved model designed to overcome the limitations of the previous model as follows [39,40]:

(24) $$Z_{c}=\rho_{0}c_{0}[1+Q-jQF]$$

(25) $$k_{c}=\frac{\omega }{c_{0}}[Q\frac{1+\left(1+Q\right)\left(1+B\right)F}{1+Q}+j\left(1+Q\left(1+B\right)\right)]$$

where *B* and *F* are coefficients that are less than 1 for very thin fibrous materials. However, as the fiber diameter increases, *B* approaches 0, whereas *F* approaches 1.

Voronina’s empirical models are based on a somewhat different approach. Instead of relying solely on airflow resistance, they also incorporate porosity, fiber diameter, and related morphological descriptors into the acoustic prediction framework [31,39,40]. In this sense, they can be considered a bridge between purely single-parameter empirical laws and later equivalent microstructure-based fluid models. Their advantage is that it is not necessary to derive the entire acoustic response solely from resistivity if useful structural data are available. However, they are not as standardized in common engineering software and are more sensitive to the appropriateness of the assumed fiber-scale geometry.

#### 3.1.8. Ramis et al. Model

The Ramis et al. model was derived for natural fiber absorbers, particularly coconut-fiber materials, by minimizing the quadratic error between measured and predicted acoustic behavior. It is useful for describing the acoustic response of bio-based fiber systems. However, it should not be considered a universal correction model for all natural fibers, as density, fiber diameter, lumen structure, and structural heterogeneity vary significantly among different plant materials [41]. The model is applied by inserting the Ramis coefficients listed in Table 1 into Equations (15) and (16).

#### 3.1.9. Modified Allard–Champoux Model

The modified Allard–Champoux model is not intended as a new universal theory, but rather as a fitting-based correction approach that incorporates impedance tube measurements and inverse-law modifications for natural materials. Its main advantage is improved agreement with experimental data within the class of materials used in parameter fitting. However, the fitted coefficients should not be extrapolated to materials with different morphologies without additional validation [42].

#### 3.1.10. Dunn–Davern Model

The Dunn–Davern model retains the Delany–Bazley regression framework but recalibrates the empirical constants for low-density cross-linked polyurethane foams. The model is useful for foam absorbers with low flow resistivity, for which mineral-wool-based coefficients are not appropriate. Its main limitation is the narrow calibration range and the need to consistently convert the attenuation and phase constants into the characteristic impedance and wavenumber formulation used in Equations (15) and (16) [31,43].

#### 3.1.11. Yoon Model

The Yoon model is useful as a comparative empirical approach for fibrous absorbers. However, before using it, the source convention needs to be verified, because Tang and Yan express the acoustic response in terms of the real and imaginary components of impedance, as well as the damping and phase constants, rather than directly in terms of the characteristic impedance and wavenumber [44].

#### 3.1.12. Wu Model

Wu′s model was developed for medium-resistivity foams and is therefore relevant when neither low-resistivity foam coefficients nor classical fibrous-material coefficients provide an adequate description. Its main limitation is the empirical calibration range and the need for consistent conversion from the original propagation-constant notation to the characteristic impedance and wavenumber notation used here [45].

#### 3.1.13. Practical Use, Advantages and Limitations of Empirical Models

Empirical models are particularly suitable for the preliminary design of absorbers, rapid comparison of material thicknesses and air-gap configurations, and situations in which airflow resistivity is the only reliably available non-acoustical parameter. Their main advantages are low computational cost, simple implementation, and straightforward integration with rigid backing conditions, cavities, and multilayer transfer-matrix formulations. For highly porous fibrous materials and selected foam absorbers, they can provide useful engineering estimates of acoustic performance.

The main differences among empirical formulations arise from their calibration datasets, mathematical expressions, and intended material classes. Consequently, their predictive accuracy depends strongly on the similarity between the investigated material and the materials used during model development. Since empirical coefficients represent fitted correlations rather than independent physical parameters, these models cannot account for the individual effects of porosity, tortuosity, pore size distribution, fiber geometry, anisotropy, or frame stiffness. Therefore, empirical models are most reliable when applied within their original calibration ranges and should be validated experimentally whenever they are used for materials or conditions outside those ranges.

### 3.2. Semi-Empirical and Equivalent-Fluid Models

Semi-empirical models and equivalent-fluid models retain the same framework of layered acoustics as empirical models, but they evaluate the acoustic response using the frequency-dependent characteristic impedance and the complex wavenumber of the equivalent fluid, according to Equations (13) and (14). Each semi-empirical model provides its own formulations for these functions based on material parameters such as porosity, airflow resistivity, high-frequency tortuosity, viscous characteristic length, thermal characteristic length, static viscous permeability, and thermal permeability [46]. Unlike empirical models, semi-empirical models have a clearer physical basis and explicitly account for the influence of the material microstructure. For rigid-frame porous materials, including glass wool, mineral wool, melamine foam, metal foam, rigid mats made of natural fibers, and many additively manufactured porous structures, equivalent-fluid models generally provide a good balance between prediction accuracy and physical interpretability. However, the rigid-frame assumption is insufficient for soft, limp, or elastic porous materials, in which the motion of the solid frame contributes to the acoustic response. In such cases, models with a flexible frame or fully poroelastic Biot models are required. The most commonly used semi-empirical equivalent-fluid models are described in the following sections.

#### 3.2.1. Zwikker–Kosten Model

The Zwikker–Kosten model describes sound propagation in porous materials idealized as bundles of rigid, cylindrical capillaries. It provides frequency-dependent effective density and bulk modulus accounting for viscous and thermal losses. The formulation is given by the equations [47]:

(26) $$\rho \left(\omega \right)=\frac{\rho_{0}}{1+\frac{2}{\vartheta }\frac{J_{1}\left(\vartheta \right)}{J_{0}\left(\vartheta \right)}}$$

(27) $$K\left(\omega \right)=\frac{\gamma P_{0}}{1+\frac{2\left(\gamma -1\right)}{C\vartheta }\frac{J_{1}\left(C\vartheta \right)}{J_{0}\left(C\vartheta \right)}}$$

where *J*_0_ and *J*_1_ are the Bessel functions of the first kind, *η* is the dynamic viscosity of air, *γ* is the specific heat ratio, *P*_0_ is the ambient static pressure, $\vartheta =\mu \cdot \sqrt{-j}$ is the complex frequency parameter, *μ* = (*ωρ*_0_*r^2^*/*η*)^1/2^ is the dimensionless viscous parameter, *r* is the capillary radius, and $C=\sqrt{Pr}$, where *Pr* is the Prandtl number of air.

The model is based on an idealized cylindrical capillary representation of the pore space, and is therefore mainly applicable to capillary-like porous materials. Its validity is limited for materials with complex or irregular pore structures such as foams, fibrous media, or granular materials, where additional validation is required.

#### 3.2.2. Attenborough Model

The Attenborough model extends rigid-frame porous formulations to fibrous and granular materials by introducing pore-geometry and tortuosity parameters. It is defined by open porosity *ϕ*, airflow resistivity *σ*, high-frequency tortuosity *α*_∞_, pore-shape factor *b*, and the auxiliary viscous parameter *χ*′ = [8*ρ*_0_*ηα*_∞_/(*σϕ*)]^1/2^*/b*. Here, *Pr* denotes the Prandtl number of air. The effective density and bulk modulus are given by [31,48]:

(28) $$\rho \left(\omega \right)=\frac{\rho_{0}\alpha_{\infty }}{\phi \left[1-\frac{2}{\chi^{\prime }\sqrt{-j}}T\left(\chi^{\prime }\sqrt{-j}\right)\right]}$$

(29) $$K\left(\omega \right)=\frac{\gamma P_{0}}{\phi \left[1+\frac{2\left(\gamma -1\right)}{{Pr}^{1/2}\chi^{\prime }\sqrt{-j}}T\left({Pr}^{1/2}\chi^{\prime }\sqrt{-j}\right)\right]}$$

where *T* = *J*_1_/*J*_0_ is the Bessel function ratio. The model is applicable to rigid fibrous and granular materials when the pore-shape approximation is valid. Its limitation is that real porous media may exhibit irregular, anisotropic, or multimodal pore structures that cannot be represented by a single pore-shape parameter.

#### 3.2.3. Wilson Model

Wilson described viscous and thermal dissipation using relaxation processes. Instead of directly using the JCA set of characteristic lengths, the model introduces characteristic relaxation times. The dynamic density and dynamic bulk modulus are given by Wilson’s original relaxation-matched formulation, as summarized in the review literature [31,49]:

(30) $$\rho \left(\omega \right)=\rho_{\infty }\frac{{\left(1+j\omega \tau_{vor}\right)}^{1/2}}{{\left(1+j\omega \tau_{vor}\right)}^{1/2}-1}$$

(31) $$\left(\omega \right)=K_{\infty }\frac{{\left(1+j\omega \tau_{ent}\right)}^{1/2}}{{\left(1+j\omega \tau_{ent}\right)}^{1/2}+\gamma -1}$$

where *ρ*_∞_ is the high-frequency density parameter, *K*_∞_ is the high-frequency bulk-modulus parameter, *τ_vor_* is the vorticity-mode relaxation time, and *τ_ent_* is the entropy-mode relaxation time. For consistency with the equivalent-fluid formulation, the asymptotic parameters can be related to JCA-type parameters as follows [31,49]:

(32) $$\rho_{\infty }=\frac{\alpha_{\infty }\rho_{0}}{\phi }$$

(33) $$\tau_{vor}=\frac{2\rho_{0}\alpha_{\infty }}{\phi \sigma }$$

(34) $$K_{\infty }=\frac{\gamma P_{0}}{\phi }$$

This model is compact and suitable for cases where acoustic behavior can be described using relaxation times. It is particularly useful for describing the transition between viscous behavior at low frequencies and inertial behavior at high frequencies. Its limitation is that relaxation times must be determined or estimated, since they are not directly measurable quantities such as porosity, airflow resistance, or characteristic lengths.

#### 3.2.4. Johnson–Champoux–Allard Model

The Johnson–Champoux–Allard (JCA) model is the contemporary engineering reference for rigid-frame porous materials. It combines the Johnson–Koplik–Dashen model dynamic density with the Champoux–Allard thermal correction and requires five transport parameters: open porosity *ϕ*, airflow resistivity *σ*, high-frequency tortuosity *α*_∞_, viscous characteristic length *Λ*, and thermal characteristic length *Λ′*. The governing equivalent-fluid relations are formulated as follows [50,51]:

(35) $$\rho \left(\omega \right)=\frac{\alpha_{\infty }\rho_{0}}{\phi }\left[1+\frac{\sigma \phi }{j\omega \rho_{0}\alpha_{\infty }}\sqrt{1+j\frac{4\alpha_{\infty }^{2}\eta \rho_{0}\omega }{\sigma^{2}\Lambda^{2}\phi^{2}}}\right]$$

(36) $$K\left(\omega \right)=\frac{\gamma P_{0}/\phi }{\gamma -\left(\gamma -1\right){\left[1-j\frac{8\kappa }{\Lambda^{\prime 2}C_{p}\rho_{0}\omega }\sqrt{1+j\frac{\Lambda^{\prime 2}C_{p}\rho_{0}\omega }{16\kappa }}\right]}^{-1}}$$

where *κ* is the thermal conductivity of air, and *C_p_* is the specific heat capacity at constant pressure. Different but algebraically equivalent forms of the second equation appear in the literature, depending on the choice of thermal function and sign convention. However, the essential physical aspect is that the JCA model captures both viscous and thermal boundary-layer dynamics in a frequency-dependent manner. The model is highly effective for open-cell foams, fibrous blankets, metallic foams, and natural porous media with sufficiently rigid frames, for which solid-phase motion can be neglected [50,51,52].

The main strengths of the JCA model are physical transferability, availability in commercial and academic software, and compatibility with inverse parameter identification procedures. Its limitations are most apparent at low frequencies, where the asymptotic behavior of the real part of the dynamic density is not accurately captured, and in soft-frame materials, where frame motion becomes significant. JCAPL-type corrections can improve the low-frequency rigid-frame description, whereas Biot-type models are required when frame motion is significant [31,46,52,53,54,55,56].

#### 3.2.5. Johnson–Champoux–Allard–Lafarge Model

The Johnson–Champoux–Allard–Lafarge (JCAL) model extends the JCA model by introducing the static thermal permeability $k_{0}^{\prime }$, thereby improving the low-frequency thermal behavior [53]. A representative expression is given as follows:

(37) $$K\left(\omega \right)=\frac{\gamma P_{0}/\phi }{\gamma -\left(\gamma -1\right){\left[1-j\frac{\phi \kappa }{k_{0}^{\prime }C_{p}\rho_{0}\omega }\sqrt{1+j\frac{4k_{0}^{\prime 2}C_{p}\rho_{0}\omega }{\kappa \Lambda^{\prime 2}\phi^{2}}}\right]}^{-1}}$$

With the same effective density *ρ*(*ω*) as the JCA model and a more accurate description of dynamic compressibility, the JCAL model is a suitable equivalent-fluid approach when high rigid-frame accuracy is required over a wide frequency band, particularly in research-grade material characterization and optimization. It is now widely used for natural-fiber panels, advanced foams, and inverse characterization studies. The main trade-off is the introduction of an additional parameter, which makes inverse fitting more sensitive to over-parameterization when the data quality is poor [52,53,57,58,59,60].

#### 3.2.6. Johnson–Champoux–Allard–Pride–Lafarge Model

The Johnson–Champoux–Allard–Pride–Lafarge (JCAPL) model introduces low-frequency corrections on both the viscous–inertial and thermal branches. Compared with the JCAL model, it requires additional low-frequency parameters, including the static viscous permeability *k*_0_, static thermal permeability $k_{0}^{\prime }$, static viscous tortuosity *α*_0_ and static thermal tortuosity $\alpha_{0}^{\prime }$. Using the notation adopted in the literature, the JCAPL model is described by the equations [31,53]:

(38) $$\rho \left(\omega \right)=\frac{\rho_{0}}{\phi }\alpha_{\infty }\left[1+\frac{\eta \phi }{j\omega \rho_{0}k_{0}\alpha_{\infty }}\;\tilde{\alpha }\left(\omega \right)\right]$$

(39) $$\tilde{\alpha }\left(\omega \right)=1-P+P\sqrt{1+\frac{M}{2P^{2}}j\frac{\omega \rho_{0}k_{0}\alpha_{\infty }}{\eta \phi }}$$

(40) $$M=\frac{8k_{0}\alpha_{\infty }}{\phi \Lambda^{2}}$$

(41) $$P=\frac{M}{4\left(\alpha_{0}/\alpha_{\infty }-1\right)}$$

(42) $$K\left(\omega \right)=\frac{\gamma P_{0}}{\phi }\cdot \frac{1}{\gamma -\left(\gamma -1\right){\left[1+\frac{\kappa \phi }{j\omega \rho_{0}k_{0}^{\prime }C_{p}}\;{\tilde{\alpha }}^{\prime }\left(\omega \right)\right]}^{-1}}$$

(43) $${\tilde{\alpha }}^{\prime }\left(\omega \right)=1-P^{\prime }+P^{\prime }\sqrt{1+\frac{M^{\prime }}{2\cdot {P^{\prime }}^{2}}j\frac{\omega \rho_{0}k_{0}^{\prime }C_{p}}{\kappa \phi }}$$

(44) $$M^{\prime }=\frac{8k_{0}^{\prime }}{\phi {\Lambda^{\prime }}^{2}}$$

(45) $$P^{\prime }=\frac{M^{\prime }}{4\left(\alpha_{0}^{\prime }-1\right)}$$

The JCAPL model is the most complete member of the Johnson–Champoux–Allard family considered here. It is suitable for research-grade characterization when the full parameter set is available. It should not be used as a default engineering model when the additional low-frequency parameters cannot be independently justified.

#### 3.2.7. Horoshenkov–Swift Model and Related Pore Structure Models

The Johnson–Champoux–Allard family represents the pore network using transport parameters. An alternative approach relates these transport parameters to pore-size statistics within a Horoshenkov-type framework. In the work by Suo et al. [61], the three-parameter approximation expresses the viscous characteristic length, thermal characteristic length, viscous permeability, thermal permeability, and tortuosity in terms of porosity *ϕ*, median pore size *s*, and the standard deviation of pore size *σ_s_*:

(46) $$\Lambda =s\;e^{-\frac{5}{2}{\left(\sigma_{s}log2\right)}^{2}}$$

(47) $$\Lambda^{\prime }=s\;e^{\frac{3}{2}{\left(\sigma_{s}log2\right)}^{2}}$$

(48) $$\;\;\;k_{0}=\frac{s^{2}\phi }{8\alpha_{\infty }}e^{-6{\left(\sigma_{s}log2\right)}^{2}}$$

(49) $$k_{0}^{\prime }=\frac{s^{2}\phi }{8\alpha_{\infty }}e^{6{\left(\sigma_{s}log2\right)}^{2}}$$

(50) $$\alpha_{\infty }=e^{4{\left(\sigma_{s}log2\right)}^{2}}$$

These relations correspond to a three-parameter approximation of the JCAL model [61].

The SS (Slanted Parallel Identical Uniform Slits) model idealizes the pore space as a set of identical, uniformly slanted parallel slits. It is suitable for slit-like or channel-like structures where a slit geometry provides a reasonable approximation. Lashgari et al. define the single-slit complex density *ρ*(*ω*) and complex compressibility *C*(*ω*) as [62]:

(51) $$\rho \left(\omega \right)=\frac{\rho_{0}}{H\left(\lambda \right)}$$

(52) $$C\left(\omega \right)=\frac{1}{\gamma P_{0}}\left[\gamma -\left(\gamma -1\right)H\left(\lambda \sqrt{Pr}\right)\right]$$

For a parallel-sided slit, the parameter *H*(*λ*) is expressed as follows:

(53) $$H\left(\lambda \right)=1-\frac{tanh\left(\lambda \sqrt{-j}\right)}{\lambda \sqrt{-j}}$$

The corresponding bulk material quantities and layer functions are [62]:

(54) $$\rho_{b}\left(\omega \right)=\frac{\alpha_{\infty }}{\phi }\rho \left(\omega \right)$$

(55) $$C_{b}\left(\omega \right)=\phi C\left(\omega \right)$$

(56) $$k\left(\omega \right)=\omega \sqrt{\rho_{b}\left(\omega \right)C_{b}\left(\omega \right)}$$

(57) $$z_{c}\left(\omega \right)=\frac{1}{\rho_{0}c_{0}}\sqrt{\frac{\rho_{b}\left(\omega \right)}{C_{b}\left(\omega \right)}}$$

(58) $$z\left(t\right)=z_{c}\left(\omega \right)coth\left[-jk\left(\omega \right)t\right]$$

(59) $$R\left(t\right)=\frac{z\left(t\right)-1}{z\left(t\right)+1}$$

(60) $$\alpha \left(t\right)=1-{\left|R\left(t\right)\right|}^{2}$$

where *λ* is the slit-related dimensionless viscothermal parameter, *t* is the sample thickness, *z*(*t*) is the normalized input impedance, *R*(*t*) is the reflection coefficient, and *α*(*t*) is the normal-incidence sound absorption coefficient.

The NUPSD model represents non-uniform pore size distributions using Padé-type correction functions. Lashgari et al. provide the Padé approximation for the normalized complex bulk density as [62]:

(61) $$\frac{\rho_{b}\left(\omega \right)}{\rho_{0}}=\frac{\alpha_{\infty }}{\phi }\left[1+\frac{F_{\rho }\left(\varepsilon_{\rho }\right)}{\varepsilon_{\rho }^{2}}\right]$$

(62) $$F_{\rho }\left(\varepsilon \right)=\frac{1+a_{\rho 1}\varepsilon_{\rho }+a_{\rho 2}\varepsilon_{\rho }^{2}}{1+b_{\rho 1}\varepsilon_{\rho }}$$

The Padé approximation for the complex bulk compressibility is given as [62]:

(63) $$C_{b}\left(\omega \right)={\left(\gamma P_{0}\right)}^{-1}\left[\gamma -\left(\gamma -1\right){\left(1+\frac{F_{C}\left(\varepsilon_{C}\right)}{\varepsilon_{C}^{2}}\right)}^{-1}\right]$$

(64) $$F_{C}\left(\varepsilon \right)=\frac{1+a_{C1}\varepsilon_{C}+a_{C2}\varepsilon_{C}^{2}}{1+b_{C1}\varepsilon_{C}}$$

where $\varepsilon_{\rho }$, $\varepsilon_{C}$, $a_{\rho 1}$, $a_{\rho 2}$, $b_{\rho 1}$, $a_{C1}$, $a_{C2}$, $b_{C1}$ are the auxiliary parameters. The NUPSD model further defines the following relationships:

(65) $$\sigma^{\prime }=\mu /k_{0}^{\prime }$$

(66) $$\Lambda =\bar{r}exp\left[\frac{-5{\left(\beta log2\right)}^{2}}{2}\right]$$

(67) $$\Lambda^{\prime }=\bar{r}exp\left[\frac{3{\left(\beta log2\right)}^{2}}{2}\right]$$

where $\bar{r}$ is the mean pore radius.

The Horoshenkov-type, SS, and NUPSD models are suitable when detailed information on the pore geometry or pore size distribution is available. Their main limitation is the reliance on idealized geometric or statistical descriptions of the pore network. Prediction accuracy may decrease for materials exhibiting strong anisotropy, multimodal pore size distributions, pore constrictions, or disconnected pores.

#### 3.2.8. Practical Use, Advantages and Limitations of Semi-Empirical Models

Semi-empirical and equivalent-fluid models provide a compromise between empirical regressions and full poroelastic or numerical models. Their main advantage is that they use measurable or identifiable non-acoustical parameters, such as porosity, airflow resistivity, tortuosity, characteristic lengths, static permeability and pore-size statistics. Consequently, they offer greater physical interpretability and better transferability than single-parameter empirical models when the material can be approximated as a rigid-frame porous medium.

The main limitation of these models is their dependence on reliable input parameters and on the validity of the assumed pore geometry. JCA and JCAL models are suitable for many rigid-frame foams, fibrous layers and granular materials if *ϕ*, *σ*, *α*_∞_, *Λ*, *Λ*′ and, for JCAL, $k_{0}^{\prime }$ are known or reliably identified. Wilson’s model is useful when relaxation times provide a compact description of the transition region. Attenborough and Zwikker-Kosten-type formulations are physically interpretable for idealized pore geometries. Horoshenkov-type models are attractive when pore-size statistics are directly measurable. SS and NUPSD models are appropriate when slit-like or non-uniform cylindrical-pore assumptions are physically justified [31,47,62,63].

For soft, limp or elastic absorbers, the rigid-frame assumption becomes insufficient. In such cases, the equivalent-fluid description should be replaced by limp-frame or Biot-type poroelastic models. Resonant structures, microperforated panels, membrane absorbers and locally resonant metamaterials require additional resonant or numerical models because their absorption is not governed solely by distributed viscous and thermal losses in a porous network.

### 3.3. Poroelastic and Moving-Frame Models

Rigid-frame and equivalent-fluid models assume that the solid skeleton of the absorber is motionless. This assumption is not sufficient for soft open-cell foams, flexible fibrous blankets, nanofiber nonwovens, automotive trim systems, porous layers bonded to vibrating panels, or cellular and metallic foams in which frame inertia, frame deformation or structural coupling contributes to the acoustic response. In such cases the absorber must be treated as a coupled two-phase medium: the pore fluid provides inertia, compressibility and viscous–thermal dissipation, whereas the solid frame provides inertia and, in the full poroelastic formulation, elastic stiffness and structural damping.

From a modeling point of view, three limiting descriptions are especially important. A rigid-frame equivalent-fluid model is sufficient when the frame motion is negligible. A limp-frame model retains frame inertia but neglects frame stiffness. A full Biot-type poroelastic model accounts for both frame inertia and frame elasticity. This hierarchy is practically important because a physically over-parameterized poroelastic model may be less robust than a simpler equivalent-fluid model if the elastic constants, loss factors and coupling parameters of the frame are not known with sufficient accuracy.

#### 3.3.1. Biot Theory

Biot’s theory is the fundamental continuum framework for wave propagation in fluid-saturated porous elastic solids. In the low-frequency formulation, Biot introduced coupled equations for the motion of the elastic frame and the saturating fluid, including inertial coupling and viscous interaction between both phases [54]. The theory predicts two compressional waves and one shear wave. The first compressional wave is mainly associated with in-phase motion of the solid frame and the pore fluid, whereas the second compressional wave is associated with out-of-phase motion and strong viscous attenuation. The experimental observation of this second compressional wave by Plona confirmed a central prediction of Biot’s model [56].

Biot later extended the theory to higher frequencies by introducing a correction for the breakdown of Poiseuille flow in the pores and for the transition toward boundary-layer-dominated viscous flow [55]. In sound-absorbing materials, this full poroelastic description is needed when frame elasticity, structural resonance, bonded-layer effects, double-porosity effects, or coupled airborne/structure-borne transmission significantly modifies the absorption spectrum. For thick mineral wool layers, rigid foams or rigid natural-fiber boards, the solid frame is often sufficiently immobile and an equivalent-fluid model is usually adequate. For lightweight fibrous blankets, nanofiber layers and soft trim materials, the frame may move while remaining too soft to support significant elastic stresses; in that regime the limp-frame approximation is often more appropriate than the full Biot model [64,65,66].

#### 3.3.2. Biot–Allard Model

The Biot–Allard formulation combines Biot-type frame motion with an equivalent-fluid description of the pore fluid. Bécot and Jaouen reformulated dissipative Biot theory so that the fluid-related coupling coefficients can be expressed through the dynamic volumic mass and dynamic bulk modulus of the porous medium, whereas the elastic frame remains described by its independent elastic and damping properties [64]. This is useful for sound-absorbing materials because the viscous–thermal part can be supplied by a JCA, JCAL or other equivalent-fluid model, while the frame deformation is retained only when it is physically relevant.

For reference, the normal-incidence equivalent-fluid relations used by Jin et al. for a homogeneous layer backed by a rigid wall can be written as [30]:

(68) $$\alpha =1-{\left|\frac{Z_{N}-\rho_{0}c_{0}}{Z_{N}+\rho_{0}c_{0}}\right|}^{2}$$

(69) $$Z_{N}=-jZ_{c}cot\left(k_{c}t\right)$$

where *Z_N_* is the normal acoustic impedance.

The characteristic impedance and complex wavenumber are obtained from the effective density and dynamic bulk modulus using Equations (13) and (14). For a JCA-type pore fluid, the dynamic density and dynamic bulk modulus may be written in the form used by Jin et al. [30]:

(70) $$\rho \left(\omega \right)=\frac{\alpha_{\infty }\rho_{0}}{\phi }\left[1+\frac{\sigma \phi }{j\omega \rho_{0}\alpha_{\infty }}\sqrt{1+\frac{4j\omega \alpha_{\infty }^{2}\mu \rho_{0}}{\sigma^{2}\Lambda^{2}\phi^{2}}}\right]$$

(71) $$K\left(\omega \right)=\frac{\gamma p_{A}}{\phi }{\left[\gamma -\left(\gamma -1\right){\left(1+\frac{8\mu }{j\omega \Lambda^{\prime 2}Pr\rho_{0}}\sqrt{1+\frac{j\omega \Lambda^{\prime 2}Pr\rho_{0}}{16\mu }}\right)}^{-1}\right]}^{-1}$$

where *p_A_* is the atmospheric pressure, and *µ* is the dynamic viscosity of air.

In a full Biot–Allard calculation, Equations (68)–(71) do not by themselves represent the entire poroelastic model. Rather, they provide the dissipative pore-fluid functions that are coupled to the elastic frame equations. The required frame parameters include at least frame density, Young’s modulus, Poisson’s ratio and structural loss factor. This formulation is therefore particularly appropriate for soft foams, fibrous lining materials, porous composites, vibrating perforated plates and multilayer systems where airborne and structure-borne transmission interact [64]. Its main practical limitation is parameter uncertainty: when frame elastic data are unavailable or poorly identified, a rigid-frame or limp-frame equivalent-fluid approximation may be more reliable.

#### 3.3.3. Limp-Frame Model

The limp-frame model is a controlled simplification of Biot theory in which the solid frame has inertia but negligible elastic stiffness. The skeleton is therefore allowed to move with the acoustic field, but it is assumed not to sustain significant shear or bulk elastic stresses. This model is important for lightweight fibrous blankets, nanofiber nonwovens, flexible textile layers, soft porous sheets and some automotive trim materials. Song and Bolton used transfer matrix measurements to distinguish the effective properties of limp and rigid porous layers [65], while Sakamoto et al. showed that, for nanofiber nonwovens, the dominant limp-frame parameters can often be reduced to bulk density and airflow resistivity [66].

Using the notation adopted by Jin et al., the effective limp density is [30]:

(72) $$\rho_{limp}\left(\omega \right)=\frac{\rho^{\prime }\left(\omega \right)\rho_{t}-\rho_{f}^{2}}{\rho_{t}+\rho^{\prime }\left(\omega \right)-2\rho_{f}}$$

(73) $$\rho_{t}=\rho_{b}+\phi \rho_{f}$$

where *ρ_limp_*(*ω*) is the effective density of the coupled moving medium, *ρ′*(*ω*) is the rigid-frame effective density of the pore fluid, *ρ_t_* is the effective moving mass density, *ρ_b_* is the bulk density of the porous material, and *ρ_f_* is the density of the saturating fluid.

The corresponding propagation constant and characteristic impedance are then calculated as for an equivalent fluid, as shown in the limp-frame formulation used by Sakamoto et al. [66]:

(74) $$\gamma_{limp}=j\omega \sqrt{\frac{\rho_{limp}\left(\omega \right)}{K\left(\omega \right)}}$$

(75) $$Z_{limp}=\sqrt{\rho_{limp}\left(\omega \right)K\left(\omega \right)}$$

where *γ_limp_* is the limp-frame propagation constant, and *Z_limp_* is the limp-frame characteristic impedance.

The limp model is close in computational cost to an equivalent-fluid model, but it accounts for frame inertia. It should not replace the full Biot formulation when elastic frame resonances, shear stiffness, strong panel coupling or structural transmission dominate the acoustic response.

### 3.4. Resonant Analytical Models for Special Absorbers

Resonant absorbers are governed mainly by acoustic inertance, cavity compliance, perforation or neck losses and, in flexible systems, structural vibration of panels or membranes. Their absorption is usually concentrated around one or more resonance frequencies, which makes them especially useful for low-frequency noise control where conventional porous layers would require excessive thickness. They cannot be described solely by distributed porous equivalent-fluid laws. Instead, their impedance must include the local resonance mechanism.

#### 3.4.1. Maa’s Model for Microperforated Panels

For a microperforated panel (MPP) backed by an air cavity, the normalized surface impedance is the sum of perforation resistance, perforation inertance and rear-cavity reactance. The compact Maa–MPP form used in the cited MPP literature can be written as [67,68,69]:

(76) $$z_{MPP}\left(\omega \right)=r_{MPP}+j\omega m_{MPP}-jcot\left(k_{0}D\right)$$

(77) $$r_{MPP}=\frac{32\mu t}{\phi_{p}\rho_{0}c_{0}d^{2}}\left[{\left(1+\frac{k_{p}^{2}}{32}\right)}^{1/2}+\frac{\sqrt{2}}{8}k_{p}\frac{d}{t}\right]$$

(78) $$m_{MPP}=\frac{t}{\phi_{p}c_{0}}\left[1+{\left(9+\frac{k_{p}^{2}}{2}\right)}^{-1/2}+0.85\frac{d}{t}\right]$$

(79) $$k_{p}=\frac{d}{2}\sqrt{\frac{\omega \rho_{0}}{\mu }}$$

(80) $$\phi_{p}=\frac{\pi d^{2}}{4b^{2}}$$

where *z_MPP_* is the normalized surface impedance of the MPP–cavity system, *r_MPP_* is the normalized resistance, *m_MPP_* is the acoustic inertance term, *d* is the perforation diameter, *t* is the panel thickness, *b* is the center-to-center spacing of the holes in a square lattice, *φ_p_* is the perforation ratio, *k_p_* is Maa’s perforation parameter, *D* is the depth of the rear cavity, and *μ* is the dynamic viscosity. The Maa model is suitable for acoustically rigid microperforated panels with small perforations. When the base panel is flexible, the panel vibration must be included separately.

#### 3.4.2. Maa–Flex Model for Flexible Perforated Panels

For finely perforated wooden, polymeric, or composite panels, vibration of the base plate may contribute significantly to the acoustic response and should therefore be included as an additional mechanical branch. In normalized impedance form, the flexible-panel branch can be expressed as follows [69]:

(81) $$z_{flex}\left(\omega \right)=\frac{1}{\rho_{0}c_{0}}\left[R_{m}+j\left(\omega m_{s}-\frac{K_{s}}{\omega }\right)\right]$$

(82) $$z_{MF}\left(\omega \right)={\left(z_{MPP}^{-1}+z_{flex}^{-1}\right)}^{-1}$$

(83) $$z_{s}\left(\omega \right)=z_{MF}\left(\omega \right)-jcot\left(k_{0}D\right)$$

(84) $$K_{s}={\left(2\pi f_{r}\right)}^{2}m_{s}$$

where *z_flex_* is the normalized mechano-acoustic impedance of the flexible plate, *z_MF_* is the normalized impedance of the flexible perforated panel without the rear cavity, *m_s_* is the surface mass density, *R_m_* is the mechanical resistance per unit area, *K_s_* is the equivalent surface stiffness, and *f_r_* is the resonance frequency of the dominant panel-vibration mode.

The Maa–Flex model should be preferred over the rigid Maa–MPP model when measured or expected base-panel vibration contributes to the absorption curve, provided that the source-specific Maa–Flex formulation or an explicitly stated flexible-panel impedance correction is used.

#### 3.4.3. Helmholtz Resonators

Helmholtz resonators combine the acoustic mass of a neck with the compliance of a cavity. For resonators with neck and cavity sections, Romero-Garcia et al. express the resonator impedance using the effective wavenumbers and characteristic impedances of the neck and cavity [70]:

(85) $$Z_{HR}=jZ_{n}\frac{A-tan\left(k_{n}l_{n}\right)tan\left(k_{c}l_{c}\right)}{Atan\left(k_{n}l_{n}\right)+tan\left(k_{c}l_{c}\right)}$$

(86) $$A=\frac{Z_{c}}{Z_{n}}$$

The effective length correction is introduced as [70]:

(87) $$\Delta l=\Delta l_{1}+\Delta l_{2}$$

where *Z_HR_* is the resonator impedance, *l_n_* and *l_c_* are the neck and cavity lengths, *k_n_* and *k_c_* are the corresponding effective wavenumbers, *Z_n_* and *Z_c_* are the corresponding characteristic impedances, and Δ*l* is the total end correction.

A single Helmholtz resonator is intrinsically narrow-band; broadband absorption requires coupled, graded, parallel or distributed resonator systems, often combined with porous layers or MPP sheets [70,71].

#### 3.4.4. Panel and Membrane Absorbers

A panel or membrane absorber can be represented as a damped mass-spring branch. The surface mass density determines the inertial component, the rear cavity or boundary conditions determine the stiffness, and material or viscous losses determine the resistance. Using the same normalized impedance convention as in the Maa–Flex branch, the panel impedance is [69]:

(88) $$z_{p}\left(\omega \right)=\frac{1}{\rho_{0}c_{0}}\left[R_{p}+j\left(\omega m_{p}-\frac{K_{p}}{\omega }\right)\right]$$

where *m_p_* is the surface mass density of the panel or membrane, *R_p_* is the mechano-acoustic resistance, and *K_p_* is the equivalent surface stiffness.

For a panel backed by an air cavity of depth *D*, the total normalized surface impedance is [69]:

(89) $$z_{s}\left(\omega \right)=z_{p}\left(\omega \right)-jcot\left(k_{0}D\right)$$

where *D* is the rear-cavity depth.

The corresponding mass-spring resonance frequency is [69]:

(90) $$f_{p}=\frac{1}{2\pi }\sqrt{\frac{K_{p}}{m_{p}}}$$

Panel and membrane absorbers are useful for low-frequency absorption, but they are sensitive to boundary conditions, pre-tension, damping, surface mass density and structural stiffness.

### 3.5. Computational Frameworks and Inverse Methods

Computational frameworks do not replace constitutive models of porous or resonant media. Instead, they assemble them into layer models, finite-element simulations, periodic-cell calculations or inverse identification workflows. In a review article, these methods should therefore be presented as implementation and validation tools rather than as additional material laws.

#### 3.5.1. Transfer Matrix Method

The transfer matrix method (TMM) is an efficient framework for one-dimensional, normally incident, layered absorbers. It combines the characteristic impedance, complex wavenumber and thickness of each layer into a global layer response. This makes it particularly suitable for impedance tube simulations, porous layers with air gaps, microperforated panel systems, limp-frame layers and porous–resonant hybrids. Song and Bolton used a transfer matrix approach to identify the characteristic impedance and wavenumber of limp and rigid porous materials [65], while Sakamoto et al. applied TMM to calculate the absorption of nanofiber nonwovens from limp-frame effective properties [66]. The method is less suitable when the sample exhibits strong lateral periodicity, finite-size effects, oblique incidence, spatial nonlocality or complex three-dimensional resonator geometry.

#### 3.5.2. Finite-Element and Full-Wave Pressure Formulations

Finite-element and related full-wave methods are required when the acoustic field cannot be reduced to a one-dimensional layer problem. They are especially important for Helmholtz-resonator arrays, acoustic metamaterials, locally resonant unit cells, 3D-printed periodic structures, metallic foams, perforated panels, and coupled vibroacoustic systems. Yang et al. used finite-element simulations to visualize the sound-pressure distributions at peak absorption frequencies of adjustable parallel Helmholtz acoustic metamaterials and to support the interpretation of broadband low-frequency absorption [71]. Khandel et al. used finite-element simulations together with experiments to evaluate additively manufactured metallic foams with constant and graded porosity Voronoi structures [72]. Full-wave methods provide detailed fields and geometry-level insight, but they require reliable boundary conditions, material parameters and mesh convergence checks.

#### 3.5.3. Boundary, Time-Domain, and Periodic Numerical Models

Periodic finite-element models and Bloch–Floquet-type unit-cell calculations are useful for metamaterials, sonic crystals, lattice absorbers and 3D-printed periodic porous structures. They allow the designer to connect microgeometry with effective acoustic response, resonance positions, band-gap behavior and spatial field localization. Such models are particularly valuable when a porous layer is no longer acoustically homogeneous, or when absorption is governed by local resonances rather than by distributed viscous–thermal losses. For practical absorber design, periodic simulations should be validated against finite-sample models and impedance tube or reverberation room measurements because edge conditions, manufacturing tolerances and mounting details can shift the predicted absorption peaks.

#### 3.5.4. Inverse Identification and Hybrid Optimization

Inverse identification is used when non-acoustical parameters, such as porosity, airflow resistivity, tortuosity, characteristic lengths, frame density, elastic constants or resonator-loss parameters, are not all available from independent measurements. In this approach, the parameters of a selected model are adjusted until the calculated absorption curve matches experimental data. The method is useful for JCA, JCAL, limp-frame, Biot–Allard, MPP and hybrid porous–resonant models, but it is sensitive to non-uniqueness: different parameter sets can produce very similar absorption curves. Therefore, the identified parameters must be constrained by physically realistic bounds and, whenever possible, supported by independent measurements. Recent studies on nanofiber media, natural-fiber absorbers, aerogels, and porous metallic structures show that hybrid workflows combining experiments, inverse identification, and numerical simulations are often effective for complex absorbers [30,66,70,71,72].

## 4. Comparison of Model Predictions with Experimental Data

An important methodological caveat should be kept in mind when interpreting the comparative literature. A model cannot be considered superior simply because it is mathematically more sophisticated. Rather, its suitability depends on whether its fundamental assumptions are consistent with the physical properties of the material system in question and whether its input parameters can be determined with sufficient reliability. Accordingly, this study first compares selected models within the same modeling class and then evaluates models across different classes. The evaluation is based primarily on peer-reviewed studies in which normal-incidence sound absorption curves are compared with experimental measurements.

### 4.1. Comparison of Empirical Models

Maderuelo–Sanz [73] investigated the sound absorption properties of cellulose acetate samples using both experimental measurements and empirical prediction models. A total of nine samples with varying physical properties, including thickness, bulk density, surface density, porosity, and tortuosity, were analyzed in this study. The measured frequency-dependent acoustic response was compared with predictions from the Delany–Bazley (DBM), Garai–Pompoli (GPM), Miki (MKM), and Komatsu (KM) models. The comparison between experimental results and model predictions revealed varying levels of agreement among the evaluated approaches. It was found that the average prediction errors for the investigated cellulose acetate samples ranged from 0.8% to 8.7%. Among the evaluated models, the MKM and DBM approaches provided the most accurate predictions, with maximum mean errors below 3%. In contrast, the GPM and KM models exhibited larger deviations from the experimental data over a broader frequency range, particularly for certain sample configurations. The superior performance of the MKM and DBM models was particularly evident at low frequencies, where the MKM model showed the closest agreement with the measured results. These findings indicate that the MKM and DBM models provided the best agreement for the investigated cellulose acetate samples and test conditions.

Garai and Pompoli [37] developed the Garai–Pompoli empirical model for predicting the sound absorption properties of polyester fiber materials. The model was developed using a dataset of 38 polyester fiber mats with fiber diameters ranging from 18 to 48 μm. The predicted sound absorption spectra were compared with experimental measurements and with the Delany–Bazley and Dunn–Davern models. Among the evaluated models, the MI model showed the closest agreement with the measured sound absorption coefficient over a wide frequency range, especially at low and high frequencies. The average prediction error was reduced by approximately 34% compared to the Delany–Bazley model and by 20% compared to the Dunn–Davern model. These results indicate that the proposed MI model provides a more accurate description of the acoustic performance of polyester fiber absorbers while maintaining a simple formulation suitable for engineering applications.

The improved empirical Komatsu model [38] for predicting the sound absorption properties of fibrous materials, based solely on airflow resistivity, was developed and validated using measurements on 15 glass wool samples and 9 mineral wool samples covering a wide range of bulk density and airflow resistivities. Compared to the Delany–Bazley and Miki models, the proposed formulation introduces logarithmic terms that better capture the experimentally observed behavior of the characteristic impedance and propagation constant. The proposed formulation shows better agreement with the measured sound absorption. This improvement is particularly evident for materials with very low or very high airflow resistivity, where conventional models are less accurate. It can be concluded that the Komatsu model extends the applicability of empirical predictions while maintaining a simple formulation based solely on airflow resistivity.

### 4.2. Comparison of Semi-Empirical Models

**Figure 1:**
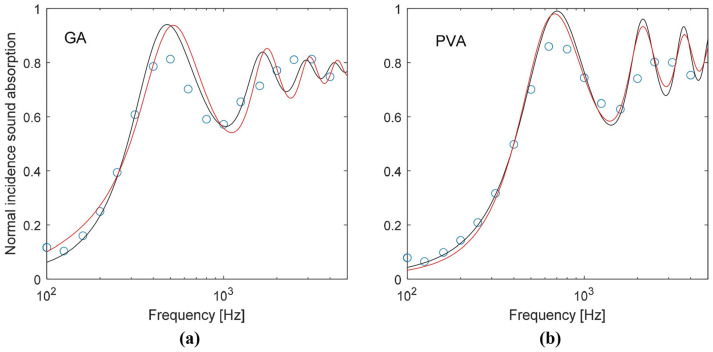
Comparison of measured (blue circles) and best-fit normal-incidence sound absorption coefficients obtained using the JCA (black curve) and JCAL (red curve) models: (**a**) AS0_GA sample; (**b**) AS1_PVA sample (reproduced from [63] under the Creative Commons Attribution License (CC BY 4.0)).

Liuzzi et al. [63] investigated the sound absorption properties of porous panels made from almond shell waste bound with gum arabic (AS0_GA) and polyvinyl acetate (AS1_PVA). The acoustic behavior of the materials was simulated using the JCA and JCAL equivalent-fluid models, with non-acoustic parameters obtained from direct measurements and parameter inversion. As shown in Figure 1, both models exhibited very good agreement with the measured normal-incidence sound absorption coefficients and accurately reproduced the main absorption peaks and overall spectral trends. The JCA model provided slightly better agreement than the more complex JCAL model, suggesting that the additional parameter in JCAL did not significantly improve the predictions. High tortuosity values, associated with the layered porous structure of the composites, contributed to increased sound absorption at low frequencies. These results confirm the suitability of equivalent-fluid models for predicting the sound absorption properties of bio-based porous materials.

**Figure 2:**
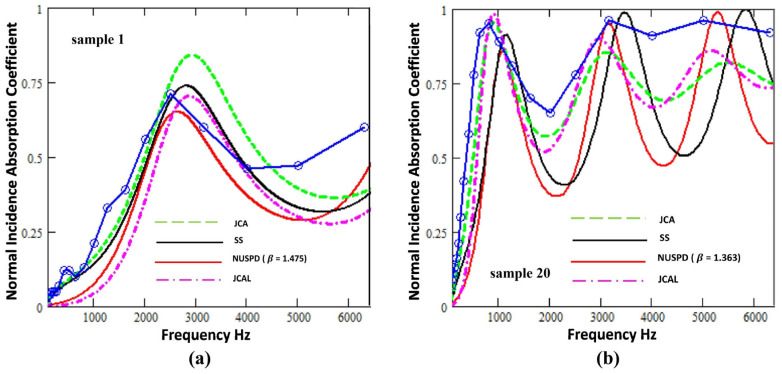
Comparison of measured absorption coefficient spectra (blue curve) for two representative wood chip samples with predictions of four modeling approaches: (**a**) sample 1 (*t* = 20 mm, *ρ_b_* = 176 kg·m^−3^, *σ* = 5880 N·s·m^−4^, *ϕ* = 72.3%, *α*_∞_ = 2.2, *Λ* = 240 μm, *Λ′* = 380 μm); (**b**) sample 20 (*t* = 50 mm, *ρ_b_* = 204 kg·m^−3^, *σ* = 5410 N·s·m^−4^, *ϕ* = 63.3%, *α*_∞_ = 1.96, *Λ* = 75 μm, *Λ′* = 350 μm) (reproduced with permission [62]. Copyright 2024, Applied Acoustics, Elsevier).

Lashgari et al. [62] investigated the sound absorption of glued laminated particleboards made of beech and Indian wood residues, whose microstructure is highly heterogeneous and is governed by particle size, porosity, bulk density, and airflow resistivity. Figure 2 shows two representative samples used in the analysis: sample 1, characterized by a coarse structure and low airflow resistivity, and sample 20, characterized by a finer structure and higher airflow resistivity. The study compares four modeling approaches: the slanted parallel identical uniform slits (SS) model, the Johnson–Champoux–Allard (JCA) model, the Johnson–Champoux–Allard–Lafarge (JCAL) model, and the statistical non-uniform pore size distribution (NUPSD) model. The JCA and JCAL models use effective macroscopic parameters identified via inverse fitting from finite element method (FEM)-based impedance tube simulations, while the JCAL model additionally accounts for thermal permeability effects. The SS model provides a simplified geometrical representation of the pore structure, while the NUPSD model uses a statistical log-normal pore size distribution to reduce parameter dependence. Overall, the study shows that model accuracy strongly depends on microstructural complexity, with JCAL providing the best agreement with experimental data, especially for finer structures.

Using a simplified three-parameter approximation of the JCAL model, Suo et al. [61] investigated the effects of fiber diameter, material density, and zeolite content on the sound absorption of porous glass-fiber materials. Increasing density and decreasing fiber diameter reduced pore size and improved sound absorption, particularly at low and medium frequencies, with the optimal pore size ranging from 60 to 80 µm. The model predicted the acoustic behavior of homogeneous fibrous materials with errors below 6%, while larger deviations (up to 13%) were observed for glass fiber-zeolite composites due to microstructural effects not fully captured by the simplified approach. Overall, the results suggest that the three-parameter JCAL model is an effective tool for predicting the acoustic properties of porous fibrous materials.

### 4.3. Comparison of Poroelastic and Moving-Frame Models

**Figure 3:**
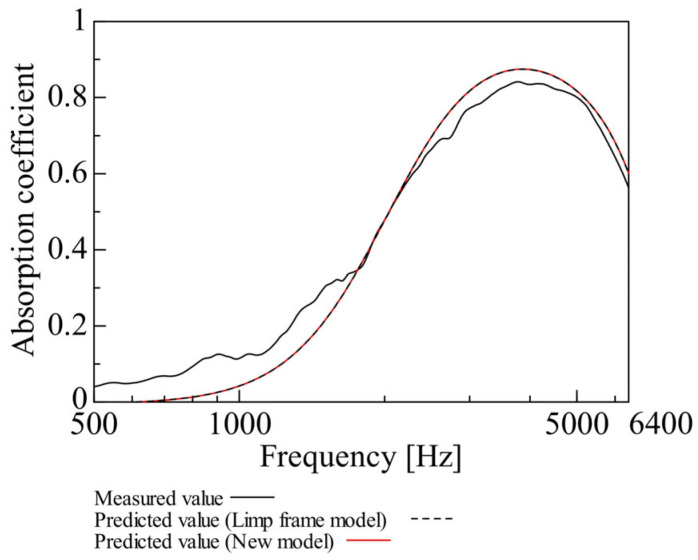
Comparison of measured and predicted sound absorption coefficients of a nonwoven nanofiber composite (sample A, *s* = 0.2 g·m^−2^, *t* = 60 µm, *b* = 303 kg·m^−3^) (reproduced from [66] under the Creative Commons Attribution License (CC BY 4.0)).

Sakamoto et al. [66] investigated nonwoven nanofiber composites composed of electrospun polyvinylidene fluoride nanofiber layers deposited on spunbond PET substrates, resulting in laminated structures with systematically varied structural parameters. The nanofibers were produced by electrospinning and partially impregnated into the PET substrate to a depth of approximately 20 µm. The samples (A–F) differed primarily in nanofiber surface density, substrate thickness, bulk density, porosity, and flow resistivity. These macroscopic parameters were used as input data for a simplified prediction model based on the Limp framework. Figure 3 compares the measured sound absorption coefficient at normal-incidence with predictions from both the original Limp framework model and the proposed simplified model. As shown in Figure 3, both models accurately reproduced the overall frequency-dependent trend for the samples. The predicted peak absorption and corresponding resonance frequency were very close to the experimental results. Minor deviations were observed at low frequencies, where sound absorption was slightly underestimated, and at high frequencies, where it was slightly overestimated. Importantly, the simplified model performed nearly identically to the full formulation of the Limp framework while reducing parameter complexity. This confirmed that bulk density, flow resistivity, and porosity were sufficiently dominant descriptors for reliable acoustic prediction of nanofiber nonwoven composites.

### 4.4. Comparison Across Empirical, Semi-Empirical, and Poroelastic and Moving-Frame Model Families

Taban et al. [74] investigated the sound absorption properties of natural coconut fiber composites and compared experimental measurements with predictions from three empirical porous-material models: Delany–Bazley, Miki, and Johnson–Champoux–Allard (JCA). The study examined samples with thicknesses of 25, 35, and 45 mm at constant density to evaluate the effect of sample geometry on acoustic performance. A comparison between measured and predicted normal-incidence sound absorption coefficients across the entire frequency range was performed. All models reproduced the general trend of increasing absorption with frequency, which is typical for fibrous porous materials. A clear thickness dependence was observed, with thicker samples showing higher absorption, particularly at low frequencies, where the 45 mm sample showed the best acoustic performance. The Delany–Bazley and Miki models slightly overestimated the absorption coefficient at higher frequencies, particularly for thinner samples. In contrast, the JCA model showed the closest agreement with the experimental data over the entire frequency range. The largest deviations between predictions and measurements were observed for the 25 mm samples at low frequencies, while the agreement improved with increasing sample thickness. These results indicate that, although all models reproduced the main acoustic trends, the JCA model provided the most accurate predictions due to its more detailed representation of the material’s microstructural parameters.

The sound absorption properties of natural coir fiber were also experimentally investigated and compared with predictions obtained using the Delany–Bazley (DB), Miki, Allard–Champoux (AC), Johnson–Champoux–Allard (JCA), and Johnson–Champoux–Allard–Lafarge (JCAL) models in studies [31,74]. Three coir fiber specimens with thicknesses of 20, 30, and 45 mm and corresponding bulk densities of 130, 153, and 159 kg·m^−3^ were analyzed. For the 20 mm thick specimen, all models reproduced the general trend of the frequency-dependent sound absorption coefficient. However, noticeable deviations were observed across parts of the investigated frequency range, resulting in relative mean errors ranging from 33% to 43%. In contrast, for the 45 mm thick specimen, substantially better agreement between experimental and predicted values was achieved, with the JCA and JCAL models exhibiting the lowest relative mean errors of 10% and 14%, respectively, while the Delany–Bazley and Miki models showed larger deviations of 25% and 30%, respectively. These results suggest that prediction accuracy improves with increasing specimen thickness and bulk density. Furthermore, the more physically based JCA and JCAL models provided a more reliable description of the acoustic behavior of natural coir fiber compared to the empirical models.

The same set of prediction models previously applied to natural coir fibers was used to evaluate the acoustic performance of kenaf specimens [31,75] investigated with varying thicknesses (10–40 mm) and two bulk densities (150 and 200 kg·m^−3^). The results indicate that the model accuracy strongly depends on material configuration. Thinner and denser kenaf samples generally exhibited larger prediction errors, reaching up to 50–55% relative mean error, whereas increasing thickness significantly improved agreement between model predictions and experimental measurements. It was found that the best overall performance was obtained for the 40 mm thick specimen with a bulk density of 150 kg·m^−3^, where the JCA and JCAL models achieved the lowest errors (7% and 13%, respectively). Empirical models (Delany–Bazley and Miki) consistently exhibit higher deviations compared to more physically grounded approaches. In general, the results confirm that the JCA- and JCAL-based formulations provide a more reliable description of the acoustic behavior of kenaf, similarly to natural coir fiber samples, while also reinforcing the general trend that prediction accuracy improves with increasing sample thickness.

A similar trend was also observed for spherical steel slag materials [31,76], for which the same set of acoustic prediction models was applied to samples with particle diameters ranging from 0–0.71 mm to 1.4–2.0 mm and layer thicknesses of 50 mm and 100 mm. Consistent with the findings for coir and kenaf fibers, the JCA and JCAL models provided the closest agreement with experimental data and exhibited relative mean errors of less than 10% in all investigated cases. In contrast, the Delany–Bazley and Miki empirical models exhibited significantly larger deviations between experimentally measured and predicted values of the sound absorption coefficient. For the coarse slag fraction, increasing the layer thickness from 50 mm to 100 mm significantly improved prediction accuracy for all models. The results also suggest that the particle size and the associated bulk density influence model performance, with the finest and densest fraction generally exhibiting lower prediction errors compared to the coarser fractions.

### 4.5. Comparison of Resonant Analytical Models

**Figure 4:**
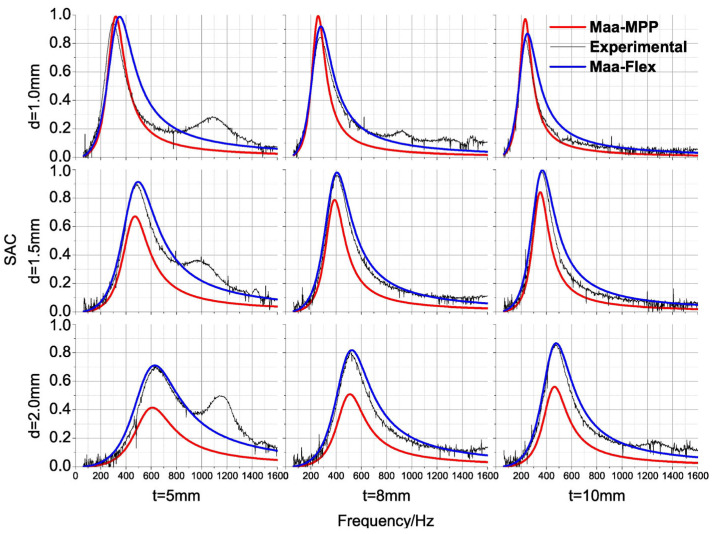
Comparison of experimental and theoretical sound absorption coefficients for perforated panels with different perforation diameters (*d*) and panel thicknesses (*t*) at a constant hole spacing of 8 mm (reproduced from [69] under the Creative Commons Attribution License (CC BY 4.0)).

The study [69] investigated the sound absorption properties of finely perforated wooden panels using two theoretical models: the Maa–MPP model and the extended Maa–Flex model. Experimental and numerical results were compared with model predictions, as shown in Figure 4. The Maa–MPP model was used as a basis for describing Helmholtz-type resonance phenomena in the perforated structure. In contrast, the Maa–Flex model additionally incorporated the effects of panel vibrations, taking into account the mechanisms of coupled mass–spring vibrations. Both models reproduced the general trends in acoustic response, but the Maa–MPP model systematically underestimated absorption outside the main resonance region. It can be concluded that the Maa–Flex model shows better agreement with the experimental data because it captures the effects of panel vibrations visible in Figure 4. The Maa–Flex model is therefore considered more accurate and physically complete for predicting the acoustic properties of finely perforated wooden panels.

**Figure 5:**
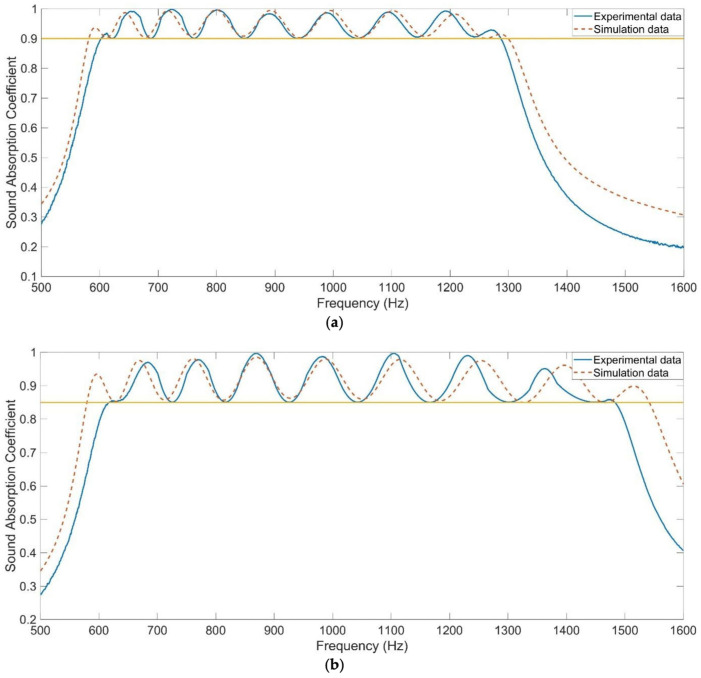
Experimental and finite-element simulated sound absorption coefficients of the optimized APH−AM (adjustable parallel Helmholtz acoustic metamaterial) samples. (**a**) Target with α > 0.90; (**b**) target with α > 0.85. The yellow horizontal lines indicate the target absorption thresholds (*α* = 0.90 in Figure 5a and *α* = 0.85 in Figure 5b). (reproduced from [71] under the Creative Commons Attribution License (CC BY 4.0)).

Yang et al. [71] presented an experimentally validated tunable Helmholtz-type acoustic metamaterial for broadband low-frequency sound absorption. The structure consisted of a parallel multi-chamber configuration of Helmholtz resonators with individually adjustable rear-cavity lengths, enabling precise tuning of resonance behavior. The sound absorption performance was investigated using a finite element model of coupled resonators and was compared with experimental measurements. A very strong agreement between simulations and experiments was observed across the entire frequency range, as clearly shown in Figure 5. The model was shown to accurately capture the shift, distribution, and coupling of resonance peaks induced by geometric adjustments. In addition, both numerical and experimental results confirmed broadband absorption arising from the coupling of multiple resonances, with absorption coefficients exceeding 0.9 within 602–1287 Hz and above 0.85 up to 1482 Hz. The findings highlight the high predictive reliability of the finite element approach for the design of tunable acoustic metamaterials.

## 5. Comparison by Material Class and Model Recommendations

This section is organized according to the material classes considered in the reviewed studies. For each class, representative studies are summarized, the main conclusions regarding the prediction of sound absorption are discussed, and the relative suitability of various modeling approaches is evaluated.

### 5.1. Polyurethane and Melamine Foams

Open-cell polyurethane foam represents one of the clearest cases in which rigid-frame equivalent-fluid models outperform single-parameter empirical formulas.

In a study by Samaei et al. [59] focusing on pure polyurethane foam and kenaf-reinforced hybrid polyurethane composites, experimental absorption spectra were compared with predictions obtained using the Delany–Bazley and JCA models. The authors reported that the JCA model reproduced both the spectral profile and the peak position significantly more accurately than the Delany–Bazley model for both pure and optimized composite foams. The deviation between the experimental data and the JCA predictions remained small across the main frequency range, whereas the Delany–Bazley model was not able to capture the mid-frequency behavior and inaccurately predicted the sound absorption coefficient peak.

The sound absorption properties of polyurethane foam were also compared with predictions from five models (Delany–Bazley, Miki, Allard–Champoux, JCA, and JCAL) in [31] for a material with a thickness of 25 mm and a bulk density of 40 kg·m^−3^. The JCA model provided the closest agreement with the experimental data, followed by the JCAL model. In contrast, the empirical Delany–Bazley and Miki models exhibited larger deviations, while the Allard–Champoux model also showed comparatively lower prediction accuracy. Overall, the results further support the conclusion that physically based JCA-type formulations provide a more reliable description of the acoustic behavior of polyurethane foams than empirical approaches.

The sound absorption performance of melamine foam specimens with a thickness of 20 mm and a bulk density of 8.4 kg·m^−3^, as well as 29.4 mm and 8.8 kg·m^−3^, was compared with predictions obtained using the Delany–Bazley, Miki, Allard–Champoux, JCA, and JCAL models [31,77,78]. The JCAL model provided the lowest error for the 20 mm specimen (11%), while the lowest errors (12%) for the 29.4 mm specimen were obtained for the Delany–Bazley, JCA, and JCAL models. The results indicate that the JCA- and JCAL-type formulations provide consistently accurate predictions across both configurations, whereas the empirical models exhibit a stronger dependence on specimen thickness and density. The maximum relative mean errors reached up to 27% for the Allard–Champoux model, whereas the JCA- and JCAL-based formulations remained below 20% in all cases.

### 5.2. Mineral and Glass Wool

Mineral wool and glass wool remain the primary focus of the Delany–Bazley, Miki, and Komatsu models, as these materials fall within the original calibration range of empirical models for fibrous materials.

**Figure 6:**
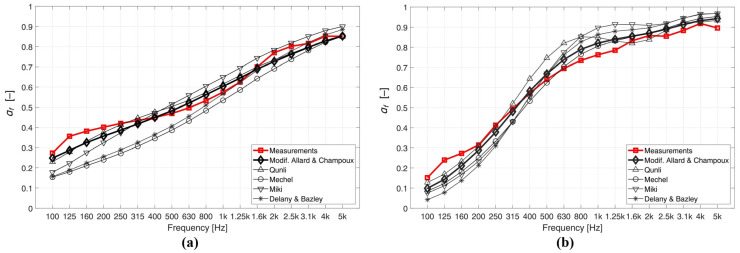
Comparison of experimental and predicted sound absorption coefficients: (**a**) mineral wool MW151, *t* = 60 mm, *ρ_b_* = 151.4 kg·m^−3^; (**b**) mineral wool with fleece MW100, *t* = 50 mm, *ρ_b_* = 100.2 kg·m^−3^ (reproduced with permission [79]. Copyright 2024, Archives of Acoustics, Polish Academy of Sciences, Committee on Acoustics).

The sound absorption behavior of mineral wool materials was investigated in [79], where the accuracy of predictions obtained from the modified Allard–Champoux, Qunli, Mechel, Miki, and Delany–Bazley models was evaluated based on a comparison with experimental measurements, as shown in Figure 6. The accuracy of the predictions was assessed using the root mean square error (RMSE) between the measured and predicted sound absorption coefficients. For both mineral wool configurations, the modified Allard–Champoux model provided the closest agreement with the experimental data and achieved the lowest RMSE values of 0.0642 and 0.0432 for MW151 and MW100, respectively. For MW151, the Qunli model achieved comparable accuracy (RMSE = 0.0648), while the Miki model showed the greatest deviation (RMSE = 0.1068). For MW100, the Delany–Bazley model exhibited the highest error (RMSE = 0.0755), while the remaining models provided intermediate RMSE values. The results suggest that the modified Allard–Champoux formulation provides the most accurate prediction of the sound absorption behavior of mineral wool materials among the examined models.

The sound absorption behavior of mineral wool materials was further investigated in [31,77], where the accuracy of the Delany–Bazley, Miki, Allard–Champoux, JCA, and JCAL models was evaluated by comparing their predictions with experimental measurements. The accuracy of the predictions was assessed using the relative mean error between the measured and predicted sound absorption coefficients. For glass wool samples with thicknesses of 20 mm and 50 mm (corresponding to bulk densities of 17 and 64 kg·m^−3^, respectively), the JCAL model provided the closest match to the experimental data for the 20 mm sample and exhibited the lowest relative mean error of 9%, while the Allard–Champoux model exhibited the largest deviation (31%). For the 50 mm thick sample, the JCA model achieved the highest prediction accuracy with a relative mean error of 7%, closely followed by the Miki and JCAL models (8%), while the Delany–Bazley and Allard–Champoux models showed errors of 15%. A similar trend was observed for the denser glass wool specimen (30 mm, 128 kg·m^−3^), for which the JCAL model also yielded the lowest relative mean error of 9%.

### 5.3. Natural Fibers

Natural fibers are significantly less homogeneous than mineral wool, and this is where the limitations of single-parameter empirical laws become most evident.

Examples of comparisons of the sound insulation properties of selected natural fiber materials were already presented in Section 4.4. Coir fiber composites were examined in terms of their sound insulation properties at various thicknesses and bulk densities. The results consistently indicated that the model’s accuracy depended strongly on the material configuration, with larger differences between experimental and predicted values observed for thinner samples, while better agreement was obtained for thicker specimens. Among the evaluated models, the JCA and JCAL formulations generally provided the most accurate predictions. Similar behavior was observed for kenaf fiber materials, where prediction accuracy improved with increasing sample thickness. Relatively large deviations between simulations and measurements were reported for thinner and denser specimens, whereas these errors were substantially reduced for thicker configurations. Overall, the results indicate that physics-based poroacoustic models provide more reliable predictions than empirical approaches for natural fiber materials.

### 5.4. Clay-Based and Ceramic Materials

Clay-based and ceramic materials exhibit heterogeneous structures: some porous ceramics behave as rigid-frame porous media and can be described using equivalent-fluid models, while others exhibit resonant or granular characteristics.

**Figure 7:**
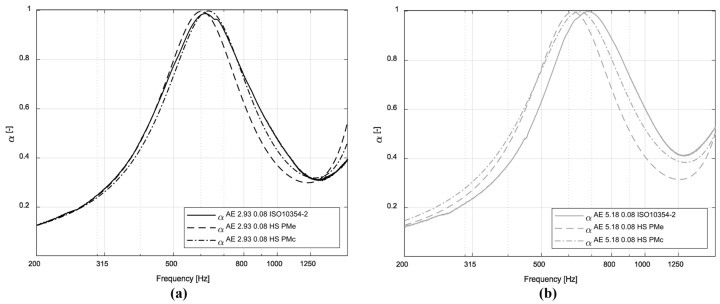
Experimental and theoretical sound absorption coefficient curves for 80 mm thick porous concrete specimens containing expanded clay: (**a**) A/C ratio = 2.93; (**b**) A/C ratio = 5.18 (reproduced from [80] under the Creative Commons Attribution License (CC BY 4.0)).

The sound absorption performance of porous concrete mixtures with A/C (Aggregate/Cement) ratios of 2.93, 3.71, and 5.18 was investigated using the Horoshenkov–Swift model [80], and the results were compared with experimental measurements. Model predictions were obtained using both experimentally determined (PMe) and corrected (PMc) macroscopic parameters. Representative results for the lowest and highest A/C ratios are shown in Figure 7. For all mixtures, the theoretical curves reproduced the characteristic absorption behavior with distinct peak and valley regions and showed good agreement with the measured data. The use of corrected parameters further improved the agreement between simulations and experiments. The best agreement was observed for the AE 2.93 and AE 3.71 mixtures, while the largest residual discrepancies remained for the AE 5.18 sample, particularly near the absorption peak. Despite the different A/C ratios, only minor variations in acoustic properties were observed among the examined mixtures. An increase in cement content was associated with a slight shift of the absorption peak toward lower frequencies, which was attributed to changes in the porous structure and the corresponding macroscopic acoustic parameters. These results indicate that the Horoshenkov–Swift model provided reliable predictions of the sound absorption behavior of porous concrete over the investigated frequency range.

### 5.5. The 3D-Printed Polymer Materials

**Figure 8:**
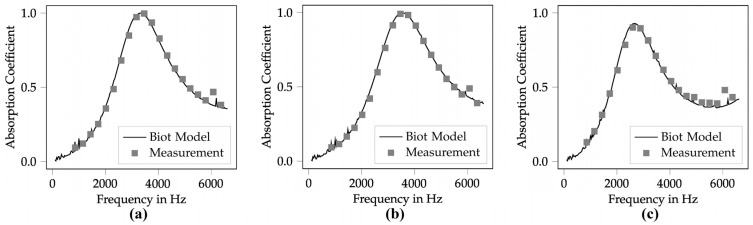
Measured and computed absorption characteristics of 3D-printed PLA samples: (**a**) Ref 1 sample: *h* = 0.2 mm, *d* = 0.5 mm, *s* = 0.3 mm, and *φ* = 90°; (**b**) Var−h sample: *h* = 0.3 mm, *d* = 0.5 mm, *s* = 0.3 mm, and *φ* = 90°; (**c**) Var−φ sample: *h* = 0.2 mm, *d* = 0.5 mm, *s* = 0.3 mm, and *φ* = 45° (reproduced from [81] under the Creative Commons Attribution License (CC BY 4.0)).

Ring and Langer [81] investigated the design, experimental characterization, and numerical modeling of 3D-printed porous sound absorbers made of polylactic acid (PLA) using fused filament fabrication (FFF). The samples consisted of periodically arranged lattices of parallel bars stacked in multiple layers, with variations in layer height *h*, bar width *d*, bar spacing *s*, and layer rotation angle *φ* (Figure 8). The aim was to correlate the controlled microstructure of the 3D-printed samples with their acoustic performance. The sound absorption coefficient was measured in an impedance tube and compared with numerical predictions. For modeling, the authors employed Biot’s poroelastic material model implemented in a finite element method (FEM) simulation of the impedance tube. The Biot parameters were identified through an inverse procedure based on the measured absorption coefficients. Good agreement was obtained between measurements and simulations for both the reference specimen (Figure 8a) and the selected geometric variants (Figure 8b,c). While variations in layer height and bar width had only a minor effect on the acoustic response, the layer rotation angle significantly influenced the flow resistivity and proved to be the most effective parameter for tuning broadband sound absorption.

Moreover, 3D-printed polymer materials are also widely used for the fabrication of micro-perforated panel (MPP) systems, for which good agreement between simulated and experimentally measured sound absorption coefficients has been reported, as further discussed in Section 5.9.

### 5.6. Metallic Foams

Metallic foams are typically rigid-frame materials, which makes JCA family models a natural choice.

The sound absorption properties of a 20 mm thick porous aluminum sample with a solid back surface, a “bottleneck” pore structure, and a porosity of approximately 60% were investigated in [82]. Experimental measurements were compared with predictions obtained using the Delany–Bazley–Miki (DBM), Attenborough, Johnson–Champoux–Allard (JCA), and Wilson relaxation models. The comparison between the measured and predicted sound absorption coefficients revealed significant differences in the predictive capabilities of the evaluated approaches. The DBM and Attenborough models exhibited poor agreement with the measured absorption spectrum over most of the investigated frequency range, while the JCA model captured the general trend but still showed noticeable deviations. The best agreement with the experimental data was achieved by the Wilson relaxation model. This model showed the closest match to the experimental results across the investigated frequency range. Its excellent performance is likely related to its ability to capture relaxation phenomena at the pore level associated with the geometry of “bottleneck-type pores”. The results suggest that conventional equivalent-fluid models may have limited ability to accurately describe porous metal structures with complex pore morphologies.

### 5.7. Fibrous Composites and Textiles

Fibrous composites and textiles occupy an intermediate position between rigid-frame porous media and limp-frame materials, which makes them particularly interesting from a modeling perspective.

Examples of comparisons between measured and predicted sound absorption properties of fibrous composites and textile materials were presented earlier in Section 4.1 and Section 4.3. Cellulose acetate materials were investigated across a wide range of thicknesses, bulk densities, porosities, and tortuosities. The results showed that empirical models generally predicted the acoustic response of these fibrous materials with relatively high accuracy. Among the evaluated approaches, the Miki and Delany–Bazley models exhibited the best agreement with experimental data, whereas the Garai–Pompoli and Komatsu models showed larger deviations within certain frequency ranges. A similar observation was made for nonwoven nanofiber composites (Figure 3), where measured sound absorption coefficients were compared with predictions based on the Limp framework and its simplified formulation. Both approaches accurately reproduced the overall frequency-dependent absorption behavior, including peak absorption values and corresponding resonance frequencies. The simplified model achieved prediction accuracy comparable to that of the full Limp formulation, while requiring fewer input parameters. These results suggest that fibrous composites and textile-based materials can often be successfully described using relatively simple acoustic models, provided that the dominant structural parameters affecting sound propagation are adequately represented.

### 5.8. Aerogels

Aerogels represent a particularly challenging class of materials due to their exceptionally high porosity and fine pore structure, which can push standard model parameters beyond the range of reliable applicability. The literature on aerogels is less developed in terms of systematic model comparisons than that on foams or fibrous materials. Nevertheless, several general trends can be identified from the available literature. Approaches based on pore size distribution, such as those inspired by Horoshenkov-type models, are attractive due to the high sensitivity of the acoustic response to pore statistics. Furthermore, composite aerogel systems can often be better described using microstructure-based equivalent fluid parameters rather than unmodified empirical coefficients.

Dasyam et al. [83] investigated the sound absorption properties of six granular silica aerogels with different particle size distributions and layer thicknesses of 25.4 mm and 50.8 mm. The experimental results were compared with predictions based on the Johnson–Champoux–Allard (JCA) framework combined with Biot’s limp porous and poroelastic formulations. For aerogel agglomerates with an average particle size larger than 100 μm, the limp porous model accurately reproduced the measured absorption spectra and provided excellent agreement with the experimental data. In contrast, for aerogels with particle sizes below 100 μm, the limp formulation systematically underestimated the low-frequency absorption peaks. Significantly better agreement was obtained using the poroelastic model, which accounts for the elastic response of the granular structure. The results demonstrate that particle size strongly influences the appropriate modeling approach. While larger aerogel agglomerates can be successfully described as limp porous media, smaller aerogel particles require poroelastic modeling to accurately capture their low-frequency sound absorption behavior.

### 5.9. Microperforated Panels (MPP)

MPP systems represent one of the most significant examples of cases where porous constitutive laws are not a suitable primary modeling tool. Examples of comparisons between the measured and predicted sound absorption properties of microperforated panels were presented earlier in Section 4.5. Using the Maa–MPP and Maa–Flex models, finely perforated wooden panels with various hole diameters and panel thicknesses were investigated, as shown in Figure 4. Both approaches successfully reproduced the general frequency-dependent absorption behavior of the investigated panels. However, the Maa–MPP model tended to underestimate the absorption coefficient outside the main resonance region. Better agreement with the experimental data was achieved using the Maa–Flex model, which additionally accounts for the effects of panel vibrations and the structural-acoustic coupling. The results suggest that incorporating panel dynamics can significantly improve the accuracy of sound absorption predictions for microperforated panel systems. In general, the Maa–Flex model appears to provide a more physically complete description of the acoustic behavior of finely perforated panels than the conventional Maa–MPP formulation.

For MPP systems, the primary modeling question is whether the panel can be considered acoustically rigid. If so, the classical Maa model, implemented within the transfer matrix method (TMM) framework, provides a suitable starting point. Otherwise, an extended formulation that accounts for the panel’s flexibility is required. Multi-layer MPP systems remain attractive because stacking multiple microperforated elements broadens the sound absorption bandwidth. However, the primary modeling framework is still based on Maa-type models combined with TMM or finite element methods (FEM), rather than porous-material approaches such as the JCA or Delany–Bazley families of models.

Liu et al. [84] investigated 3D-printed micro-perforated panel absorbers (MPPA) combined with air gaps and a porous layer to enhance sound absorption performance. The system consisted of a polymer MPPA with varying perforation ratios backed by an air cavity, and, in the multilayer configuration, an additional fibrous porous material. The acoustic response was modelled using Maa’s theory for the MPPA backed by an air gap, while the multilayer absorber was analyzed using the transfer matrix method (TMM), with the porous layer described by the Johnson–Champoux–Allard (JCA) model. Good agreement was found between model predictions and experimental measurements for both investigated configurations. The model accurately predicted the resonance peak, and the addition of the porous layer significantly broadened the sound absorption bandwidth. Similar conclusions were also obtained in [85], where good agreement between the theoretical model and experimental results was confirmed for a simplified MPPA-porous configuration. The modeling approach combined Maa’s theory for the micro-perforated panel with an equivalent fluid model for the porous layer, implemented within the transfer matrix method framework. The simulations consistently showed that the perforation ratio primarily controls the resonance frequency, while the porous backing mainly enhances broadband absorption without significantly shifting the resonance peak.

High agreement between simulated and measured sound absorption coefficients was obtained for multi-layer MPP structures [20] using Maa’s model for the panels together with finite-element simulations and experimental validation. Similarly, good agreement between calculated and measured results was achieved for multiple-layer perforated panel systems [72] using the transfer matrix method (TMM), where individual panels and air cavities were represented as cascaded acoustic transfer elements, enabling prediction of absorption performance and resonance behavior.

### 5.10. Helmholtz Resonator Panels

**Figure 9:**
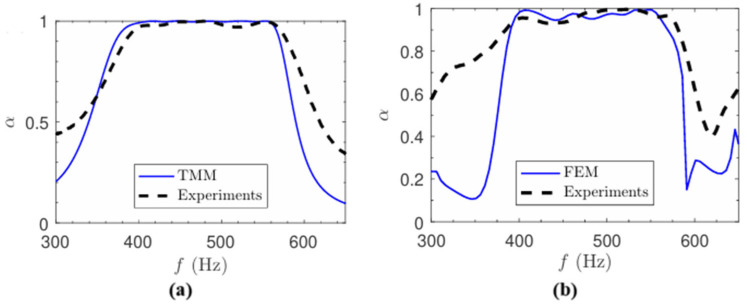
Comparison of the predicted and measured sound absorption coefficients for: (**a**) 1D impedance tube system; (**b**) 2D anechoic chamber system (reproduced from [86] under the Creative Commons Attribution License (CC BY 4.0)).

Herrero-Durá et al. [86] investigated the sound absorption performance of two-dimensional arrays of Helmholtz resonators. The system was first optimized in a one-dimensional configuration using the transfer matrix method (TMM), with the geometry of the individual resonators tuned to achieve near-perfect absorption in a selected low-frequency range. This design was then extended to a 2D periodic array and numerically analyzed using finite element method (FEM) simulations, including finite-size effects and multiple scattering. Experimental validation was performed in an impedance tube (1D case) and an anechoic chamber (2D case). As shown in Figure 9a, the 1D system exhibited almost perfect absorption in the target frequency band, with very good agreement between TMM predictions and measurements. Similarly, Figure 9b shows that the 2D array maintained high absorption performance, with strong agreement between simulations and experimental results, confirming the reliability of the modeling approach.

**Figure 10:**
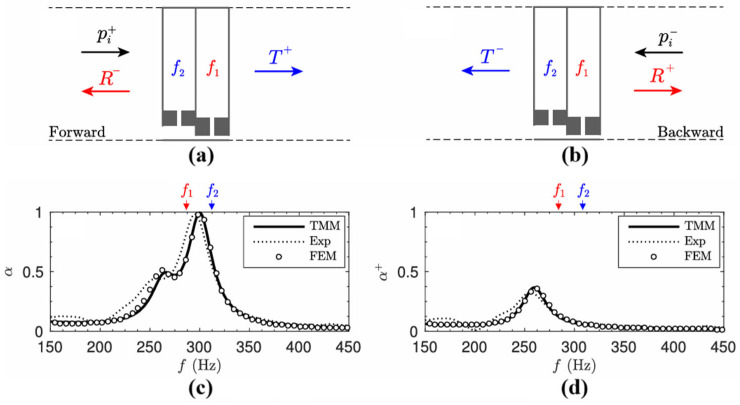
Schematic of the sub-wavelength asymmetric panel in: (**a**) forward; (**b**) reverse configuration. Sound absorption results obtained using TMM, FEM, and experiments for: (**c**) the forward configuration; (**d**) the backward configuration (reproduced from [87] under the Creative Commons Attribution License (CC BY 4.0)).

Romero-García et al. [87] investigated the design of Helmholtz-resonator-based acoustic metamaterials using the complex frequency plane. Transfer matrix and scattering matrix models were employed to identify critical coupling conditions and optimize the resonator geometry, while finite element simulations were used for numerical validation. As shown in Figure 10a–d, the study compared symmetric and asymmetric resonator configurations under forward and backward incidence. Good agreement was obtained between theoretical, numerical, and experimental absorption results, demonstrating enhanced sound absorption at the target frequencies and validating the proposed modeling approach.

A similar trend in sound absorption performance was also observed in Section 4.5, where analytical and finite element models were compared for perforated panels and Helmholtz-type acoustic systems. Yang et al. [71] presented a tunable multi-chamber Helmholtz resonator with adjustable cavity lengths, whose acoustic response was modeled using a finite element approach and validated experimentally, as shown in Figure 5. The results showed very good agreement between simulations and measurements, demonstrating the high predictive capability of the finite element model in capturing resonance shifts, coupling effects, and broadband absorption behavior.

## 6. Summary and Final Recommendations

A comparison of the above approaches to acoustic modeling suggests that the choice of model should be guided by the dominant physical mechanism, available input parameters, operating frequency range, and intended application rather than by a universal model hierarchy. The primary consideration should always be the dominant acoustic mechanism: distributed porous dissipation, frame-coupled poroelastic effects, or discrete/geometry-dependent resonance. The second consideration concerns the available input data: only basic macroscopic parameters, a complete set of transport parameters, or both transport and elastic properties of the frame. The third consideration is the design phase, including preliminary screening, parameter optimization, or final experimental or design validation. The operating frequency range represents an additional important criterion, as different physical mechanisms may dominate at low, mid, and high frequencies even within the same material class.

Based on these considerations, including frequency-dependent effects, the practical implications for model selection can be summarized as follows. The Delany–Bazley and Miki formulations are most suitable for rapid preliminary assessment of highly porous fibrous materials when airflow resistivity is the only reliably available parameter. The Garai–Pompoli and Komatsu formulations are more suitable when the materials under investigation fall within the calibration ranges of these empirical relationships. The JCA and JCAL approaches become more suitable for materials that deviate from conventional fibrous absorbers or when their transport parameters can be determined with sufficient reliability. Horoshenkov-type pore size distribution models are advantageous when pore-size statistics can be measured directly, and the assumed pore size distribution retains clear physical relevance. Limp-frame formulations are appropriate when frame inertia is relevant, but frame stiffness is negligible, whereas full Biot–Allard models are required when frame elasticity or structural coupling is significant. Finally, Maa-type microperforation models, Helmholtz-resonator approaches, TMM, and FEM are preferred in cases where sound absorption is primarily determined by geometry-dependent or resonance-controlled mechanisms.

The main practical recommendation is therefore clear: one should not automatically choose the most sophisticated model available. Instead, preference should be given to the simplest model that nevertheless captures the dominant physical phenomena within the absorber under the intended operating conditions. This represents a general principle for model selection that is applicable to fibrous media, foams, ceramics, printed structures, and resonant metamaterials.

Table 2 provides a comparative overview of the discussed acoustic model families, including their input requirements, application ranges, advantages, and limitations. Table 3 complements this overview with material-specific recommendations for model selection across different porous and resonant absorber classes. Table 4 further complements these recommendations by summarizing frequency-oriented guidance for model selection across the low-, mid-, and high-frequency ranges for each material class, illustrating how the preferred modeling approach may vary with operating frequency.

## 7. Conclusions

This review provided a critical assessment of the main modeling approaches used for predicting sound absorption in porous materials, covering empirical formulations, equivalent-fluid models, poroelastic theories, resonant approaches, and numerical methods. The comparison demonstrates that no universal modeling approach is suitable for all classes of acoustic absorbers, since predictive capability depends strongly on the dominant dissipation mechanisms, material morphology, frequency range, and availability of reliable input parameters. Empirical and semi-empirical approaches remain valuable because of their simplicity, computational efficiency, and applicability in engineering design, particularly for conventional fibrous and foam-based absorbers. Equivalent-fluid formulations provide an effective balance between physical realism and computational cost for many rigid-frame porous media, whereas poroelastic models are required when frame motion contributes significantly to the acoustic response. Resonant and metamaterial absorbers require specialized approaches capable of describing local resonance phenomena and geometry-dependent effects beyond classical porous-medium theories. The operating frequency range should also be considered when selecting a model.

The review further highlights that model accuracy depends not only on the theoretical formulation but also on the reliability of material characterization and parameter identification. Uncertainties in airflow resistivity, porosity, tortuosity, characteristic lengths, or elastic frame properties may influence prediction accuracy more strongly than the selection of a more advanced model. Therefore, the most sophisticated available model should not automatically be considered the most appropriate choice. Model selection should instead balance physical relevance, reliability of input parameters, numerical stability, and computational efficiency.

The present review provides a structured overview of model assumptions, required parameters, application ranges, and limitations, helping researchers and engineers select appropriate approaches for different material classes and design objectives. This comparison can support future experimental characterization, numerical modeling, and inverse identification by clarifying which parameters require reliable determination and which modeling assumptions require validation for specific absorber morphologies.

Future developments in acoustic material modeling are expected to involve stronger integration of multiscale simulations, image-based characterization, additive manufacturing optimization, and physics-informed machine learning methods for inverse design and parameter identification.

Overall, effective acoustic modeling should remain guided primarily by the dominant physical mechanisms governing sound attenuation rather than by model complexity alone. Robust and physically consistent model selection will therefore remain essential for the development and optimization of advanced porous materials, acoustic metamaterials, and hybrid sound-absorbing systems.

## Figures and Tables

**Table 1:** Coefficient values (*C*_1_–*C*_8_) for empirical models based on the compact characteristic impedance and complex wavenumber formulation, with source-specific conventions noted where the model does not follow Equations (15) and (16) [31,32,33].

Model	*C*_1_	*C*_2_	*C*_3_	*C*_4_	*C*_5_	*C*_6_	*C*_7_	*C*_8_
Delany–Bazley	0.0571	−0.754	0.087	−0.732	0.0978	−0.7	0.189	−0.595
Mechel (1/60 < *X* < 1)	0.0571	−0.754	0.087	−0.732	0.0978	−0.7	0.189	−0.595
Mechel (*X* ≤ 1/60)	0.0489	0.754	0.087	0.731	0.0978	0.693	0.189	0.618
Miki	0.070	−0.632	0.107	−0.632	0.160	−0.618	0.109	−0.618
Garai–Pompoli	0.078	−0.623	0.074	−0.66	0.121	−0.53	0.159	−0.571
Komatsu [Equations (18) and (19)]	0.00027	6.2	0.0047	4.1	0.0069	4.1	0.0004	6.2
Ramis et al.	0.0713	−0.8749	0.1216	−0.4520	0.2129	−0.4857	0.0997	−0.5988
Modified Allard–Champoux	0.07290	−0.66228	0.18700	−0.5379	0.0982	−0.685	0.288	−0.526
Dunn–Davern	0.114	−0.369	0.0985	−0.758	0.168	−0.715	0.136	−0.491
Yoon	0.057	−0.734	0.087	−0.732	0.0978	−0.700	0.189	−0.595
Wu	0.2090	−0.5480	0.1050	−0.6070	0.1880	−0.5540	0.1630	−0.5920

**Table 2:** Comparative overview of major acoustic model families, including input parameters, applications, advantages, and limitations.

Model Family	Required Input Parameters	Typical Application	Advantages	Limitations
Delany–Bazley/Mechel/Miki [34,35,36]	Airflow resistivity *σ*, layer thickness *t*, and boundary/load conditions (BC or *Z_L_*) are required for absorption calculations	Rapid preliminary screening of highly porous fibrous absorbers, especially mineral/glass wool and similar fibrous layers; the Mechel model extends the applicability of DB-type approaches toward lower *X* values	Very simple, computationally efficient, and directly compatible with surface-impedance or transfer-matrix calculations; the Miki model improves the positive-real behavior of the empirical formulation	Empirical and dependent on the calibration range, with limited low-frequency accuracy and no explicit representation of *φ*, *α*_∞_, *Λ*, *Λ′*, pore size distribution, or frame motion
Garai–Pompoli/Komatsu [37,38]	Primarily, airflow resistivity *σ*, layer thickness *t*, and boundary/load conditions (BC or *Z_L_*) are required for final absorption prediction	Garai–Pompoli: polyester fiber blankets; Komatsu: improved DBM/Miki-type predictions for selected fibrous products	More material-class-specific than generic DBM coefficients and able to reduce prediction errors when the material belongs to the calibration family	Still empirical and strongly dependent on the material family. The Komatsu model uses logarithmic equations and must not be included in the generic DBM-type power-law coefficient table
Other material-specific empirical variants (Voronina, Ramis, Dunn–Davern, Yoon, Wu) [39,40,41,42,43,44,45]	Airflow resistivity *σ* and structural descriptors such as porosity *φ*, fiber diameter *d*, structural parameter *Q* or fitted coefficients *C*_1_−*C*_8_ are used depending on model	Applicable to selected natural fibers, high-porosity fibrous materials, low-density polyurethane foams, and medium-resistivity foams where generic fibrous coefficients are inadequate	Better targeted to a specific morphology or material family than a universal DBM coefficient set	Not universal. Coefficients and sign conventions must be transferred carefully and should not be extrapolated to unrelated morphologies without validation
JCA [50,51]	Open porosity *φ*, airflow resistivity *σ*, high-frequency tortuosity *α*_∞_, viscous characteristic length *Λ*, and thermal characteristic length *Λ′*	Applicable to general rigid-frame porous media, including many open foams, fibrous mats, porous lattices, and rigid porous absorbers, when reliable transport parameters are available	Good balance between physical interpretability, accuracy, and implementability; explicitly includes viscous and thermal characteristic lengths	The model requires several measured or inversely identified parameters and may show reduced low-frequency accuracy because the original JCA model does not include the Lafarge-type low-frequency thermal correction
JCAL/JCAPL [53,88]	CA transport parameters plus static thermal permeability $k_{0}^{\prime }$ for JCAL; JCAPL also uses low-frequency correction parameters such as *α*_0_ and $\alpha_{0}^{\prime }$	Research-grade equivalent-fluid prediction of rigid-frame porous media when low-frequency accuracy and reliable parameter identification are required	Improves low-frequency dynamic compressibility and/or density behavior compared with the original JCA model	More demanding parameter identification; additional fitted parameters can reduce robustness if experimental data are insufficient or noisy
Zwikker–Kosten/Attenborough/Wilson [47,48,49]	Pore geometry or shape factors, capillary radius *R* or slit width *b*, porosity *φ*, flow resistivity *σ*, tortuosity *α*_∞_, and/or relaxation times *τ_vor_*, *τ_ent_*	Physically interpretable rigid-frame alternatives for capillary, fibrous, granular, and relaxation-dominated porous media	Useful for linking pore geometry, pore shape, and viscous/thermal diffusion to the effective density *ρ*(*ω*) and bulk modulus *K*(*ω*)	Each model relies on idealized pore geometry or relaxation assumptions; transfer to complex real materials requires parameter validation
Horoshenkov-type/SS/NUPSD [62,89,90]	Porosity *φ*, airflow resistivity *σ*, tortuosity *α*_∞_, and pore-size statistics, e.g., characteristic pore size and standard deviation, or non-uniform pore distribution parameters	Microstructure-informed granular, wood-chip, slit-like, and pore-distribution-dominated porous media	Physically interpretable with fewer parameters than full inversion if pore-size statistics are known or can be estimated	Accuracy depends strongly on whether the assumed pore size distribution, slit geometry, or granular morphology is valid for the tested material
Biot/Limp/ Biot–Allard [54,55,64,65]	Transport parameters plus frame density/inertia and elastic properties together with *φ*, *σ*, *α*_∞_, *Λ*, and *Λ′*; simplified limp-frame models focus mainly on inertia and equivalent density	Soft or mobile fibrous layers, trim materials, elastic foams, and nanofiber nonwovens where frame motion contributes to acoustic response	Captures frame motion, coupled air-frame phenomena, and Biot-type poroelastic effects absent in rigidframe equivalent fluids	High parameter burden for full poroelastic modeling and unnecessary for clearly rigid-frame materials; limp assumptions must be validated
Maa/Helmholtz/panel–membrane resonators [67,68,69,72,91,92]	Perforation diameter *d*, panel thickness *t*, perforation ratio *φ_p_*, cavity depth *D*, neck/cavity dimensions *S*, *L*, *V*, panel or membrane impedance *Z_m_*	Microperforated panels, Helmholtz resonators, panel/membrane absorbers, hybrid porous–resonant systems, and locally resonant metamaterials	Strong control of tuned low-frequency absorption peaks; bandwidth can be enlarged by porous backing, multiple resonators, graded cavities, or coupled resonant cells	Typically narrow-band as a single resonator and not a substitute for porous constitutive laws in the backing material; requires accurate geometry and loss modeling
TMM/FEM/BEM/inverse identification [71,72,93,94]	Constitutive model, geometry, boundary conditions, layer sequence, *Z_c_*, *k_c_*, *t*, *Z_L_* and, for inverse methods, measured *α*(*f*) or *Z_s_*(*f*) plus objective function	Multilayer systems, complex geometries, meso-heterogeneous porous media, resonant structures, and model-parameter identification	Can represent realistic assemblies, coupled porous–resonant systems, and geometry-driven acoustic mechanisms beyond closed-form single-layer formulas	Higher computational cost and strong dependence on parameter quality; inverse identification can be non-unique or unstable without constraints and validation

**Table 3:** Material-specific guidance for acoustic model selection.

Material Class	Recommended Models	Generally Unsuitable Models
PUR/melamine foams [31,43,64,65,67]	Use JCA/JCAL for open-cell rigid-frame PUR and melamine foams when *ϕ*, *σ*, *α*_∞_, *Λ* and *Λ′* are available. Use Dunn–Davern only as a foam-specific empirical screening model for low-density polyurethane foams. Use Biot–Allard only when skeleton deformation contributes to the acoustic response. Use limp-frame formulations only when frame inertia or frame motion is acoustically important.	Do not use generic fibrous empirical coefficient sets as final design models without foam-specific validation. Do not use pure Maa-type panel models for homogeneous foam layers without perforations.
Mineral/glass wool [31,34,36,38,53]	Use Delany–Bazley for rapid estimates of fibrous wool absorbers when σ is the main known material parameter. Use Miki as a modified Delany–Bazley-type empirical model for porous/fibrous materials. Use Komatsu when the logarithmic fibrous-material correction is appropriate. Use JCAL for detailed prediction when *φ*, *σ*, *α*_∞_, *Λ* and *Λ*′ are available and low-frequency thermal behavior is important.	Do not use overparameterized poroelastic models when frame elastic properties and damping cannot be identified reliably. Do not transfer foam-specific or natural-fiber empirical coefficients to mineral/glass wool without validation.
Natural fibers [31,41,65,75,76]	Use JCA/JCAL for natural coir, kenaf and date-palm fibers when required transport parameters are available. Use JCA for coconut-fiber composites when microstructural parameters are available. Use Ramis-type empirical models for coconut-fiber absorbent materials within their fitted material family. Use limp-frame formulations only when sheet mobility or frame motion affects the response.	Do not directly reuse mineral-wool, glass-wool or polyester empirical coefficients as final models for plant fibers without validation. Do not use pure resonator models when the material behaves primarily as a distributed porous fibrous absorber.
Clay-, ceramic-, concrete-, and granular mineral absorbers [31,48,62,89,90]	Use JCA/JCAL for steel slag spheres and similar rigid granular media when measured transport parameters are available. Use Attenborough-type models for rigid fibrous or granular materials where pore-shape factors are physically meaningful. Use Horoshenkov–Swift for granular materials with a pore size distribution close to log-normal. Use the three-parameter Horoshenkov–Hurrell–Groby model when characteristic pore-size statistics can be estimated.	Do not use generic single-parameter fibrous empirical laws as final models for granular, ceramic or concrete pore networks. Do not apply SS/NUPSD-type pore size distribution models without checking that their pore-geometry assumptions are valid.
3D-printed polymers [20,21,31,50,93]	Use JCA-type equivalent-fluid modeling for porous printed networks only after validating transport parameters such as *ϕ*, *σ*, *α*_∞_, *Λ* and *Λ′*. Use FEM when the printed geometry, finite-size effects or coupling cannot be reduced to a homogeneous layer. Use resonant or metamaterial models for printed resonant cavities, labyrinthine structures or locally resonant cells. Use geometry-specific modeling when 3D-printed absorbers exhibit multiple absorption peaks caused by arranged perforations, slits or cavities.	Do not choose the model solely from polymer chemistry, because the printed geometry controls the acoustic mechanism. Do not treat a resonant printed lattice as a homogeneous fibrous blanket. Do not use generic DBM/Miki laws as final models without validation of the printed pore geometry.
Metallic foams [31,50,53,88,93]	Use JCA-type equivalent-fluid modeling when metallic foam behaves as a rigid porous medium with validated dynamic permeability/tortuosity parameters. Use JCAL when the low-frequency dynamic compressibility correction is important. Use Pride/JCAPL-type corrections for more complex drag-force or low-frequency visco-inertial effects. Use FEM when structural coupling or geometry-dependent effects must be resolved numerically.	Do not use DBM or Miki as the primary final constitutive model for metallic pore morphology without validation. Do not use pure fibrous empirical laws when distributed porous losses and metallic pore morphology dominate.
Textiles/nanofibers [30,31,50,53,65]	Use JCA for thick or sufficiently stiff porous textile mats when *ϕ*, *σ*, *α*_∞_ and *Λ* are available. Use JCAL when thermal permeability or low-frequency compressibility corrections are required. Use limp-frame models for mobile fibrous sheets and nanofiber nonwovens where frame motion participates in the response. Use nanofiber-specific multiscale modeling when slip-boundary effects and nanofiber morphology dominate.	Rigid-frame assumptions are unsuitable for compliant or mobile sheets where frame motion is acoustically important. Generic fibrous empirical laws should not be used as final models without validation for the particular textile or nanofiber morphology.
Aerogels [31,50,53,83,94]	Use JCA-type modeling when aerogel behavior can be represented by validated equivalent-fluid transport parameters. Use JCAL when low-frequency thermal compressibility must be represented more accurately. Use inverse identification when measured absorption/impedance spectra are available for estimating uncertain porous parameters. Use aerogel-specific validation because aerogel microstructure may fall outside classical fibrous empirical calibration ranges.	Unmodified DBM/Miki/Komatsu laws are generally unsuitable outside their fibrous-material calibration range unless they are validated for the aerogel. Pure resonator models are unsuitable for non-resonant aerogel layers.
Microperforated panels [67,68,69,90,93]	Use Maa’s MPP model for rigid microperforated panels with small perforations. Use Maa’s extended MPP formulation for practical microperforated-panel absorber design. Use Maa–Flex when the perforated panel is flexible and panel vibration affects absorption. Use TMM for multilayer panel-cavity assemblies. Use FEM when geometry or coupling effects cannot be reduced to one-dimensional transfer matrices.	Do not use pure porous-medium laws for the perforated panel itself. Do not use DBM/Miki/JCA as if a microperforated panel were a homogeneous fibrous or porous layer.
Helmholtz panels and resonant metamaterials [10,17,70,71,93]	Use Helmholtz analytical models for resonators governed by neck inertance and cavity compliance. Use hybrid resonator models when Helmholtz resonators are mounted with microperforated panels. Use critical-coupling or resonant-metamaterial formulations for locally resonant subwavelength absorbers. Use numerical modeling when plate-type resonant metamaterial geometry or coupling is complex.	A single porous constitutive law is unsuitable for the entire resonator system. JCA/JCAL should be used only for porous fillings, backings or hybrid layers, not as the complete resonance mechanism.

**Table 4:** Frequency-oriented guidance for acoustic model selection by material class.

Material Class	Low Frequencies (20–250 Hz)	Mid Frequencies (250–2000 Hz)	High Frequencies (2–20 kHz)
PUR/melamine foams [9,25,43,53,59,64]	Use JCAL for rigid open-cell foams when transport parameters are known. Use limp-frame or Biot–Allard models when frame inertia or elasticity affects the low-frequency response.	Use JCA or JCAL as the main predictive models. For low-density cross-linked PUR, retain Dunn–Davern only as a foam-specific screening model.	Use JCA/JCAL while the foam remains an acoustically homogeneous open-pore medium. Use FEM or a microstructure-resolved model for closed-pore or meso-heterogeneous foams.
Mineral/glass wool [31,35,36,38,51,53]	Prefer JCAL for detailed low-frequency prediction. Mechel can extend a Delany–Bazley-type estimate toward lower values of *X*, but only within its stated validity range.	Use Delany–Bazley or Miki for rapid estimates when airflow resistivity is known. Use JCA/JCAL when a reliable transport-parameter set is available.	Use Miki or Komatsu within their fibrous-material calibration ranges. Use JCA/JCAL when characteristic lengths and tortuosity are known.
Natural fibers [32,41,65,74,75]	Use JCAL for rigid natural-fiber boards. Use limp-frame or Biot-type formulations for compliant mats whose skeleton moves appreciably.	Use JCA/JCAL for coir, kenaf and related fibers. Use the Ramis model only for coconut-fiber materials close to its calibration family.	Use JCA/JCAL with material-specific transport parameters. Retain empirical natural-fiber models only after validation for the actual morphology.
Clay, ceramic, concrete, and granular mineral absorbers [25,48,80,89,90]	Use JCAL or Horoshenkov-type pore size distribution models. Use FEM when strong heterogeneity, resonant cavities or frame coupling controls the response.	Use JCA/JCAL for rigid open-pore media, the Attenborough model for physically meaningful pore-shape factors, or Horoshenkov-type models for granular pore networks.	Use pore size distribution or geometry-resolved numerical models when the homogenized equivalent-fluid assumption becomes uncertain.
3D-printed polymers [20,21,50,81,93]	Use geometry-specific resonant models, TMM or FEM for printed cavities and subwavelength cells. Use an equivalent-fluid model only after transport-parameter validation.	Use JCA/JCAL for homogenizable porous lattices and FEM for periodic, anisotropic or finite-size geometries that cannot be reduced to a uniform layer.	Use periodic or full-wave FEM when the wavelength approaches the unit-cell or channel dimensions. Geometry, not polymer chemistry, should govern model selection.
Metallic foams [50,82,88,93]	Use JCAL/JCAPL for rigid open-cell foams. Use Wilson or FEM for bottleneck pores and poroelastic modeling when structural coupling is significant.	Use JCA/JCAL for validated rigid-frame morphologies. Wilson-type relaxation modeling may be superior for bottleneck-dominated structures.	Use JCA/JCAL only while homogenization remains valid. Use full-wave or FEM models when pore geometry produces local inertial or wave effects.
Textiles/ nanofibers [30,51,53,64,66]	Use limp-frame models for mobile sheets and nanofiber nonwovens. Use full Biot-type modeling when elastic frame stresses or structural resonances are non-negligible.	Use JCA/JCAL for sufficiently stiff textile mats and limp-frame models for compliant layered nonwovens.	Use nanofiber-specific multiscale models with slip-boundary corrections when fiber-scale rarefaction and morphology become important.
Aerogels [25,83,94]	Use limp-porous modeling for sufficiently large granular agglomerates. Use poroelastic modeling for finer particles when frame elasticity shifts low-frequency peaks.	Use JCA/JCAL with aerogel-specific parameters and inverse identification when transport properties cannot be measured independently.	Use JCA/JCAL only after validating homogenization. Use microstructure-resolved or hybrid numerical modeling for strongly heterogeneous particle networks.
Microperforated panels [16,20,68,69,72,84]	Use Maa’s MPP model for rigid panels and Maa–Flex when panel vibration contributes. Combine the panel model with TMM for the backing cavity.	Use multilayer Maa/TMM formulations. Model any porous backing separately with JCA/JCAL to broaden the absorption band.	Use geometry-specific Maa/viscothermal or FEM models for small perforations and multilayer panels. Use hybrid MPP–porous systems for bandwidth extension.
Helmholtz panels and resonant metamaterials [10,70,71,86,93]	Use Helmholtz impedance, critical-coupling, and FEM/TMM models. Use coupled or parallel resonators for broadband low-frequency targets.	Use graded, coupled or array-based resonator models with TMM/FEM to account for resonance interaction, multiple scattering, and finite-size effects.	Use geometry-explicit full-wave/FEM models when cell dimensions are no longer deeply subwavelength. Use JCA/JCAL only for porous fillings or backings.

## Data Availability

The original data presented in this study are openly available in [Zenodo] at https://doi.org/10.5281/zenodo.20727062 [95].

## Abbreviations

AC	Allard–Champoux Model
ANC	Active Noise Control
BEM	Boundary Element Method
DBM	Delany–Bazley Model
FDTD	Finite Difference Time Domain
FEM	Finite Element Method
GPM	Garai–Pompoli Model
ISO	International Organization for Standardization
JCA	Johnson–Champoux–Allard
JCAL	Johnson–Champoux–Allard–Lafarge
JCAPL	Johnson–Champoux–Allard–Pride–Lafarge
KM	Komatsu Model
Maa–Flex	Maa Flexible Panel Model
Maa–MPP	Maa Microperforated Panel Model
MKM	Miki Model
MPP	Microperforated Panel
NUPSD	Non-Uniform Pore Size Distribution
PNC	Passive Noise Control
RMSE	Root Mean Square Error
SS	Slanted Parallel Identical Uniform Slits
SWR	Standing Wave Ratio
TMM	Transfer Matrix Method
